# Immersed Boundary Models for Quantifying Flow-Induced Mechanical Stimuli on Stem Cells Seeded on 3D Scaffolds in Perfusion Bioreactors

**DOI:** 10.1371/journal.pcbi.1005108

**Published:** 2016-09-22

**Authors:** Yann Guyot, Bart Smeets, Tim Odenthal, Ramesh Subramani, Frank P. Luyten, Herman Ramon, Ioannis Papantoniou, Liesbet Geris

**Affiliations:** 1 Prometheus, Division of Skeletal Tissue Engineering, KU Leuven, Leuven, Belgium; 2 Biomechanics Research Unit, Université de Liège, Liège, Belgium; 3 Division of Mechatronics, Biostatistics and Sensors (MeBioS), Leuven, Belgium; 4 Biomechanics Section, KU Leuven, Leuven, Belgium; 5 Skeletal Biology and Engineering Research Center, KU Leuven, Leuven, Belgium; National University of Ireland - Galway, IRELAND

## Abstract

Perfusion bioreactors regulate flow conditions in order to provide cells with oxygen, nutrients and flow-associated mechanical stimuli. Locally, these flow conditions can vary depending on the scaffold geometry, cellular confluency and amount of extra cellular matrix deposition. In this study, a novel application of the immersed boundary method was introduced in order to represent a detailed deformable cell attached to a 3D scaffold inside a perfusion bioreactor and exposed to microscopic flow. The immersed boundary model permits the prediction of mechanical effects of the local flow conditions on the cell. Incorporating stiffness values measured with atomic force microscopy and micro-flow boundary conditions obtained from computational fluid dynamics simulations on the entire scaffold, we compared cell deformation, cortical tension, normal and shear pressure between different cell shapes and locations. We observed a large effect of the precise cell location on the local shear stress and we predicted flow-induced cortical tensions in the order of 5 pN/μm, at the lower end of the range reported in literature. The proposed method provides an interesting tool to study perfusion bioreactors processes down to the level of the individual cell’s micro-environment, which can further aid in the achievement of robust bioprocess control for regenerative medicine applications.

## Author Summary

Tissue Engineering involves the combination of cells, growth factors and biomaterials into artificial constructs which, upon implantation, can improve the healing capacity of the human body. A remaining challenge involves providing physical stimuli to individual cells, thereby guiding them towards the properties of the desired tissue type. Perfusion bioreactors try to control the local concentration of oxygen, nutrients and growth factors and mechanical stresses by varying the fluid flow. In this work, we predict the shear stress that individual cells experience at the microscopic scale, as a function of the bioreactor inlet flow velocity, by making use of the immersed boundary method. This method combines an Eulerian grid (fixed in space) with a Lagrangian grid (moving with the flow) to model the deformation of cells due to flow inside a scaffold pore. Our simulations show that the local shear stress levels on specific, realistic cell geometries are different from the shear stress levels on empty scaffolds, which are often still used as a reference. Finally, we predict and discuss the additional effect of realistic flow on other mechanical cell properties, such as its deformation and its cortical tension.

## Introduction

The culture of stem cell populations in dynamic set-ups, for example in perfusion bioreactors, holds great potential for the production of tissue engineered constructs [1]. The use of bioreactors permits automated seeding and expansion of progenitor cells [2,3], facilitating the production of clinically relevant cell populations in close systems, while maintaining their phenotype and bone forming potential [4]. Additionally, these systems can provide controlled biomechanical stimuli, such as fluid flow-induced shear stresses, that might significantly affect stem cell properties during dynamic culture in bioreactors. For example, mechanical stimuli have been associated to early stem cell lineage commitment [5] and osteogenic priming in the absence of inductive growth factors [6–8]. Moreover, they have been shown to further promote osteogenic differentiation of bone marrow, periosteum and adipose derived osteochondroprogenitor cells in the presence of osteoinductive growth factors [9–13]. Osteogenic differentiation has been linked to the magnitude of shear stress, showing dose dependent enhancement of extra-cellular matrix deposition and subsequent mineralization by the cultured cells [14–17].

In order to characterize the dynamic environment throughout cell seeded scaffolds in perfusion bioreactors, many Computational Fluid Dynamics (CFD) modeling studies have been presented in the past decade [18–22]. However, the majority of these studies considered empty scaffold geometries without incorporating a cell domain. Recently this issue was addressed by representing the growing neotissue as a porous medium in order to model the effect of neo-tissue growth on the flow profile [23–27]. Still, these models predict the local distribution of shear stress and pressure throughout a volume averaged porous domain and do not take into account the local mechanical and geometrical environment of individual cells.

Mechano-transduction of stress induced by shear flow conditions is highly localized at specific areas of the cell’s interface with its environment, such as focal adhesions, FAs [28], and primary cilia [5]. The latter have been shown to be involved in the osteogenic response of bone cells to dynamic shear flow conditions [29], as well as in remodeling of the extracellular matrix [30]. The amount of force perceived at the level of FAs as a result of external flow conditions is influenced by the cell’s mechanical properties, cell shape and the geometry of its microscopic environment, e.g. location of attachment points, and presence of extracellular matrix (ECM). In this respect, the concept of cell cortical tension has gained a renewed interest in the last years as a mediator of mechano-transduction processes [31]. Cortical tension is created by the cell itself through active acto-myosin contractility, resulting in a prestressed cytoskeleton. This self-generated stress is an essential aspect of the tensegrity theory as introduced by [32], which posits that the cytoskeleton constitutes a tensegrity structure, with tension generating cortical stress fibers as ‘ropes’ and load bearing capacity provided by other cytoskeletal elements, the substrate or the ECM. Next to additional structural integrity, the mechanically stressed state of a cell can boost the mechano-sensitivity of a cell [33]. External flow however, can also contribute to locally elevated levels of cortical tension, especially close to attachment point such as FAs. This passive source of cortical tension, as well as its importance relative to the cell-generated active tension and prestress, has not yet been investigated for perfusion cell culture systems. Therefore, a scaling gap exists between small scale i.e. ‘single cell’ and ‘neotissue/whole scaffold’ macro-scale that needs to be bridged. Computational models using realistic single cell geometries are a prime candidate for facilitating this task. Similar concepts of cross-scale model integration in order to capture mechanical interactions across scales from a whole organ to single cell level have been already described and envisaged [34,35] and have served as a paradigm for the current study focusing mostly on a scaffold-based *in vitro* process.

Computational models of cell deformation due to shear flow have been developed considering the cell as a 2D Gaussian interface [36] or a 3D linear elastic solid [23,37–47]. The latter use a mixed Lagrangian-Eulerian formulation to solve the Fluid-Structure Interaction (FSI) problem, with a coupling through continuity boundary conditions. Additional numerical methods have been recently developed for modeling fluid-flow driven solid deformations in a biomechanical context. Immersed finite element methods have been used for modeling soft tissue deformation under the influence of blood flow [47] and within the walls of the aortic root [48]. In addition cell motility and deformation through contracted channels reminiscent of microfluidic experiments were also captured using a similar method operating with a single analysis mesh for solid and fluid that was not subjected to any deformation [49]. For larger deformations, the interaction between cell and fluid has been resolved by means of the level-set method [50]. Alternatively, the Immersed Boundary Method (IBM) is able to explicitly take into account discrete entities in the cell’s cortex and, possibly, its internal cytoskeletal structure. It has been used to model the movement and deformation of vesicles, red blood cells and bacteria under flow conditions [51,52]. An FSI model for osteoblasts attached to scaffold struts was recently published [53], with a rigid single cell consisting of a half-sphere with two focal adhesion points. In the work presented in this study, more realistic cell shapes are introduced, which are not rigid but deform due to the fluid flow. Still, the cytoskeleton constitutes a highly complex, mechanoadaptive material [54–56] and its mechanical behavior differs between various temporal and spatial scales, [57,58]. Hence at present, only a strongly simplified mechanical representation of a complete attached cell is considered computationally feasible.

The main purpose of this study is to use the IBM to investigate fluid-induced mechanical stimuli on progenitor cells used for bone tissue engineering (human periosteal derived cells, hPDCs) attached to regular pore titanium scaffolds inside a perfusion bioreactor set-up. Each cell is represented by a simplified model of the cortical shell, similar to [59], supplemented with discrete Focal Adhesions (FAs) and an elastic nucleus. A multi-scale modeling approach is presented, consisting of a CFD analysis at the scaffold macroscopic (tissue) scale in order to determine suitable input boundary conditions at the microscopic scale (single cell scale) where the fluid-structure interaction is modeled by means of the IBM. The impact of the spatial location of the cells within the scaffold during flow perfusion on a number of key mechanical quantities at the cellular scale was investigated. To illustrate how (location-induced) geometrical differences might affect the biomechanical environment of single cells, three characteristic locations and corresponding cell geometries were chosen: one cell spread along the direction of the flow (A), one facing the flow (F) and one bridging between two struts (B). Furthermore, the presented model was used to assess how small clusters of cells attached to the scaffold are mechanically affected by perfusion flow. In order to investigate mechanical effects of mutual shielding [60], a ‘three cells configuration’ (T) facing the flow was investigated.

## Methods

### Immersed Boundary Method

The IBM has been developed for simulating moving, deformable membranes immersed in a fluid, based on a combination of an Eulerian and a Lagrangian approach [61]. The deformable object (the cell in this study), is represented by a discretized membrane/cortex Γ(t) and is able to move freely through the fixed Eulerian mesh Ω on which the flow is computed. The interconnection between both lattices is accomplished by means of a smoothed Dirac function δ.

In the 3D mesh Ω, the equations for incompressible Stokes flow are solved (as appropriate for the low Reynolds numbers typically encountered in bioreactors, see also in the supplementary information):

−μΔu+∇p=F, (1)

∇⋅u=0, (2)

with suitable boundary conditions which are explained in following section. In Eqs (1) and (2), ***u*** represents the fluid velocity, *p* the pressure and *μ* the viscosity. The influence of the cell boundary Γ(t) immersed in the fluid is taken into account through the distributed force density ***F*** and can be expressed as:

$$F\left(x,t\right)=\int_{\Gamma \left(t\right)}f\left(s,t\right)\delta \left(x-X\left(s,t\right)\right)ds.$$ (3)

Here, ***x*** are the Eulerian coordinates and ***X***(*s*,*t*) are the discretized cell membrane coordinates indicating the position of the membrane at time *t*. As mentioned previously, the interaction between both meshes is realized through the introduction of a Dirac function δ defined by the following continuous function of the distance *r*:

$$\delta \left(r\right)=\{\begin{array}{c} \frac{1}{4}\left(1+cos\left(\frac{\pi \left|r\right|}{2}\right)\right),\;\left|r\right|\leq 2\\ 0,\left|r\right|>2 \end{array}$$ (4)

Using Eq (4), Eq (3) can be rewritten in a discrete formulation

$$F\left(x,t\right)=\sum_{i=1}^{N}f^{i}\left(s,t\right)\delta_{h}\left(x-X_{i}\left(s,t\right)\right)ds,$$ (5)

with *N* the number of nodes of the cell membrane, *h* the Eulerian mesh size and

$$\delta_{h}\left(x\right)=\frac{1}{h^{3}}\delta \left(\frac{x}{h}\right)\delta \left(\frac{y}{h}\right)\delta \left(\frac{z}{h}\right).$$ (6)

Once the flow is computed, the membrane positions are updated using the following equation of motion:

$$\frac{dX\left(s,t\right)}{dt}=U\left(X\left(s,t\right),t\right),$$ (7)

with ***U*** the interpolated flow velocity on Γ(t) which can be expressed as follows:

$$U\left(X\left(s,t\right),t\right)=\int_{\Omega }u\left(x,t\right)\delta \left(x-X\left(s,t\right)\right)dx.$$ (8)

### Mechanical representation of a cell

**Fig 1:**
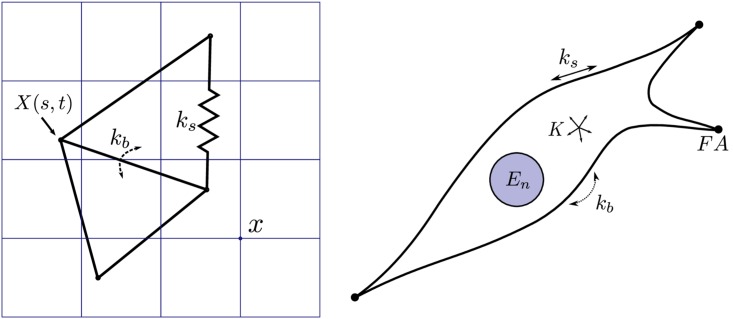
Mechanical representation of the cell. **Left**: Lagrangian elastic boundary of the cell with stretching stiffness *k*_*s*_, bending stiffness *k*_*b*_ and integration points *X*(*s*,*t*), immersed in an Eulerian lattice with positions *x*. **Right**: Mechanical representation of a cell attached to a scaffold, approximated through the use of a cortical shell model with stretching and bending stiffness (resp. *k*_*s*_ and *k*_*b*_), volume compression modulus *K* and nucleus stiffness *E*_*n*_. Discrete attachment points representing focal adhesions (*FA*) connect the cell to the rigid scaffold.

The mechanical representation of a cell in the Eulerian domain relies on the work presented in [62] where the underlying mechanisms and assumptions are discussed in detail. Briefly, the model assumes that most of the cytoskeletal material is present in a relatively thin cortical shell and that an elastic description of deformations at short timescales is adequate. Hence, the mechanical properties of this cortical shell will account for the mechanical response of the complete cytoskeleton. The immersed boundary which represents the cell is composed of a triangulated surface with a stretching stiffness *k*_*s*_ and a bending energy *k*_*b*_. Finally, the cell nucleus is represented as a submerged solid elastic sphere with Young’s modulus *E*_*n*_.–see Fig 1.

The linear spring force of node *i* for each connected node *j* at distance *d*^*ij*^ and resting length $d_{0}^{ij}$ is expressed as:

$$f_{s}^{ij}=k_{s}\left(d_{0}^{ij}-d^{ij}\right)e^{ij},$$ (9)

where ***e***^*ij*^ denotes a unit vector pointing from *i* to *j*. A moment of bending is computed between all adjacent triangles *k* and *m* with angle *θ*^*km*^ and resting angle $\theta_{0}^{km}$:

$$M_{b}^{km}=k_{b}sin\left(\theta^{km}-\theta_{0}^{km}\right).$$ (10)

A force corresponding to this moment is applied to the non-common points of each of the two triangles, and a compensating force is applied to the common edge points, ensuring that the total force on the cell remains unchanged, i.e. for two triangles with common nodes *c*_1_ and *c*_2_ and non-common nodes *l*_*k*_ and *l*_*m*_:

$$f_{c1}=f_{c2}=\;-\frac{M_{b}^{km}}{2}\left(\frac{n^{k}}{L^{k}}+\frac{n^{m}}{L^{m}}\right),$$

$$f_{l_{k}}=\frac{M_{b}^{km}}{L^{k}}n^{k},\;\;\;\;\;\;f_{l_{m}}=\frac{M_{b}^{km}}{L^{m}}n^{m}.$$ (11)

*L*^*k*^ and *L*^*m*^ are the distances from resp. node *l*_*k*_ and *l*_*m*_ to the line containing the common edge, and ***n***^*k*^ and ***n***^*m*^ are the normal unit vectors of triangle *k* and *m*. We denote the total bending force contribution of all adjacent triangle pairs of node *i* as $f_{b}^{i}$. This type of bending stiffness is commonly found in the literature for Red Blood Cell models [63]. The cell’s volume is maintained through an effective bulk modulus *K*. For this, an internal pressure *P*_*v*_ is computed based on a cell’s volume *V* and equilibrium volume *V*_0_:

$$P_{v}=K\frac{V_{0}-V}{V}\;.$$ (12)

Subsequently, a force $f_{v}^{i}=P_{v}A^{i}n^{i}$ is obtained for each node *i* with *A*^*i*^ and ***n***^*i*^ respectively the area and outward normal unit vector of each node, both of which are calculated using a discrete version of the Laplace-Beltrami operator. Furthermore, the nucleus is represented as a solid, elastic sphere with Young’s modulus *E*_*n*_ for which contact with the cortical nodes is considered Hertzian, i.e.

$$f_{n}^{i}=\{\begin{array}{cc} \frac{4E_{n}\sqrt{R_{n}}}{3}\delta_{n}^{i}{}^{3/2}e_{n}^{i} & ,\;\delta_{n}>0\\ 0 & ,\;\delta_{n}\leq 0 \end{array}$$ (13)

for node *i* indenting a nucleus with radius *R*_*n*_ and overlap distance $\delta_{n}^{i}$, and with $e_{n}^{i}$ a unit vector pointing from the nucleus center to node *i*. Discrete attachment points serve as Focal Adhesions (FAs). These points are placed outside of the fluid domain and are therefore not displaced by the fluid. Finally, the total force per node ***f***^*i*^(*s*,*t*) is computed as the sum of all aforementioned partial forces:

$$f^{i}\left(s,t\right)=f_{s}^{i}+f_{b}^{i}+f_{v}^{i}+f_{n}^{i}.$$ (14)

### Atomic Force Microscopy measurements of cell cortical stiffness

**Fig 2:**
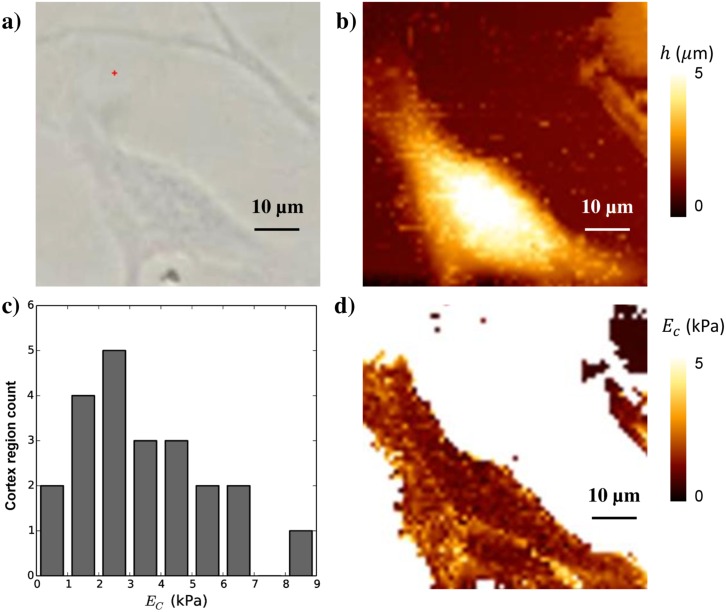
AFM measurements of the cell cortical stiffness. **a)** Optical image of the cell, **b)** AFM height map, **c)** histogram of cortical Young’s modulus *E*_*c*_ from N = 13 cells (in which in total 22 different cortex regions were sampled) **d)** Young’s modulus image.

Cell cortical stiffness was measured using Atomic Force Microscopy (AFM). Measurements were performed using a Nanowizard 3 BioScience AFM (JPK) with a working range of 100×100×15 μm mounted on the stage of an inverted microscope (Olympus 1) placed on a vibration-isolation table. A V-shaped gold-coated silicon nitride cantilever with a four-sided pyramidal tip (Budget Sensors) with a nominal tip radius *r*_tip_ of 15 nm and an opening angle *θ* of 35 degrees was used as the probe. The spring constant *k*_spring_ of the cantilever was ca. 0.3 Nm^−1^. Exact values have been calibrated using the thermal fluctuation method. Force curves have been recorded at 5 μm/s approach and retract speed, of which only the approach curves have been analyzed to arrive at the instantaneous Young’s modulus using the Sneddon model for forces > 200 pN. We neglect the information at low indentations, since according to [64], the Sneddon model is accurate at higher indentation *δ*, allowing us to extract the cortical Young’s modulus *E*_*c*_ as:

$$F=\frac{E_{c}\text{tan}\left(\theta \right)}{\sqrt{2}\left(1-\nu^{2}\right)}\delta ,$$ (15)

where *F* is the measured force. Assuming a Poisson’s ratio *ν* of 0.5 [65], we can fit this formula to the typical force-indentation curves obtained by AFM for every pixel on the cell’s surface (we use the Levenberg-Marquard algorithm in MINPACK through its python-interface provided by SciPy for curve-fitting). To extract the stiffness of the cortical layer, we select regions on the cell away from the nucleus where the average cell height is very low so that we can assume that the measured stiffness is indeed the compressive stiffness of the cell’s cortex and not dominated by effects from bending of the cortical layer or the intra-cellular fluid—see S1 Text and S2 Fig. To limit the influence of the underlying substrate, the maximal force was chosen to keep the indentation depth to less than 20–30% of the height of cortex cell thickness. The full procedure to select usable patches within the AFM stiffness maps is detailed in the supplementary information. The global average over all measured cells and all patches yields an estimated cortical stiffness *E*_*c*_ = 3.5 ± 2 kPa (Fig 2).

### Calibration of cell mechanical model

**Fig 3:**
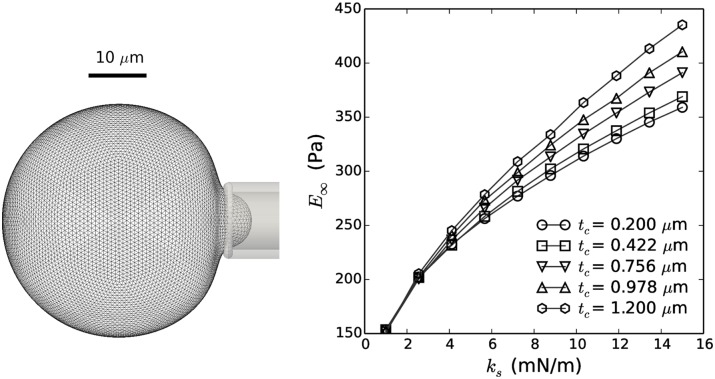
Determination of cell stiffness by means of micropipette aspiration. **Left:** Visualization of simulated micropipette aspiration experiment. **Right:** Equilibrium Young’s Modulus *E*_∞_ as a function of stretching stiffness *k*_*s*_ for varying cortical thickness *t*_*c*_. For the parameter values extracted from the AFM measurements, an equilibrium Young’s modulus of 194.7 Pa was obtained.

For a relatively thin cortical ‘sheet’, and assuming that the cortex consists out of some homogenous elastic material, the stretching stiffness *k*_*s*_ and bending energy *k*_*b*_ can be related to the cortical Young’s modulus *E*_*c*_ and the cortical thickness *t*_*c*_:

$$k_{b}=\frac{E_{c}t_{c}^{3}}{12\left(1-\nu_{c}^{2}\right)}$$ (16)

$$k_{s}=\frac{2E_{c}t_{c}}{\sqrt{3}}$$ (17)

where we usually assume the Poisson’s ratio of the actin cortex *v*_*c*_ to be close to 0.5 [66]. Having determined the effective stiffness of the cortical shell and its thickness from AFM, these equations allow us to calculate the parameters of the mechanical cell model. To evaluate our procedure, these estimated mechanical parameters can be compared to simulated Micropipette Aspiration (MA). Hereto, a simulation was set up where a spherical cell is aspirated into a thin cylindrical structure with a rounded tip and radius *R*_*p*_—Fig 3. The relationship between the applied under-pressure in the pipette and the aspirated length *L*_*p*_ of the cell expresses an effective equilibrium Young’s Modulus *E*_∞_ which can be compared to experimental values obtained using the same technique [67]:

$$\Delta P=\frac{2\pi }{3}E_{\infty }\frac{L_{p}}{R_{p}}\Phi$$ (18)

where *Φ* ≈ 2.1 is a scaling factor.

The cell’s Young’s modulus obtained by applying this procedure—see Fig 3—from the parameter values estimated from AFM measurements (Table 1) is 194.7 Pa, which compares well to measured values from MSCs; e.g. [68] report Young’s moduli in the range of 150–350 Pa.

**Table 1:** List of parameters used in study.

Parameter	Symbol	Value	Unit	Source
Viscosity	*μ*	0.001	Pa · s	Water at 293K
Bending energy cortex	*k*_*b*_	8.5e-17	Nm	AFM; Eq (15)
Cortical stiffness	*k*_*s*_	2.34e-3	N/m	AFM; Eq (16)
Bulk modulus	*K*	200	Pa	Assumption
Young’s modulus nucleus	*E*_*n*_	1000	Pa	[69]
Poisson’s ratio nucleus	*v*_*n*_	0.5	-	[69]

### Design of simulations

**Fig 4:**
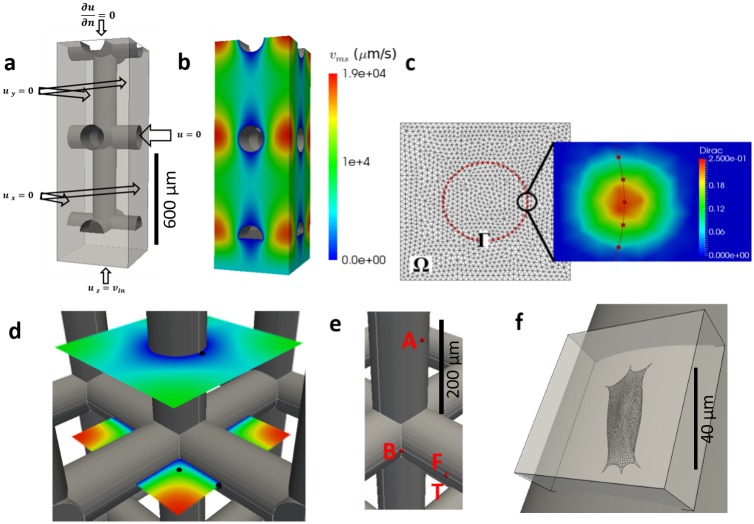
Overview of the computational domains. **a)** The computational domain and boundary conditions at the macro-scale. **b)** Computed flow velocity magnitude used as input for the micro-scale models. **c)** Immersed Boundary method representation: Ω and Γ are resp. the Eulerian and Lagrangian mesh (left), with an illustration of the smoothed Dirac distribution (right). **d)** Black dots represent the three locations where the micro-scale Dirichlet boundary conditions for flow velocity v_ib_ are extracted. **e)** Red dots represent the three locations of cells: along the flow (A), bridging (B), facing the flow (F) and ‘three cell cluster’ configuration (T). **f)** Micro-scale domain; the grey cylinder is the scaffold strut, the box is the Eulerian mesh Ω and the cell is the Lagrangian mesh Γ.

The flow profile around a single cell attached to the scaffold is computed by solving the immersed boundary problem at the scale of the investigated cell. For this purpose, the Eulerian computational domain Ω corresponds to a box of a few hundred microns wide/long containing the cell and not the whole scaffold pore—see Fig 4F.

In order to obtain the magnitude of flow velocity which is to be used as a Dirichlet boundary condition on the microscopic domain Ω, see Eqs (1) and (2), Stokes’ equation was solved on an entire scaffold pore. An inlet velocity corresponding to the bioreactor flow rate *Q*_*in*_ was set at the entrance of the pore and symmetry boundary conditions were applied on each sides of the pore–Fig 4A. Next, the calculated flow velocities *v*_*ms*_ at specific locations in the scaffold pore were used to extract suitable boundary conditions *v*_*ib*_ for the IBM problem–Fig 4D. These locations are indicated in Fig 4E and correspond to characteristic positions inside a pore: a cell on a cylindrical strut with flow parallel to the cylinder axis (A), a cell on a cylindrical strut with flow perpendicular to the cylinder axis (F), a small cluster of three interconnected cells on a cylindrical strut with flow perpendicular to the cylinder axis (T) and a single cell attached on a strut junction, forming a bridge between two perpendicular struts (B).

**Fig 5:**
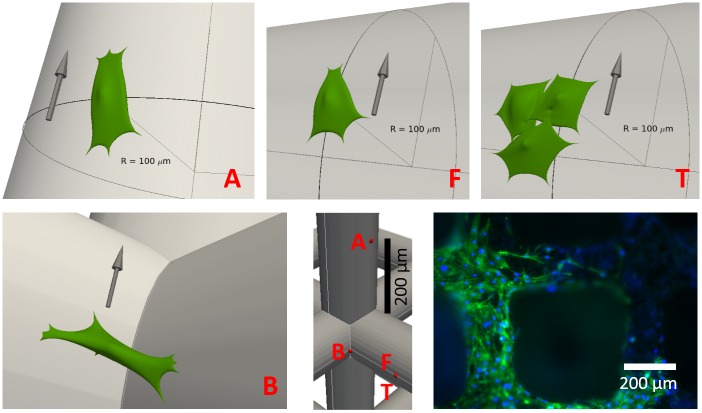
Initial representations of the four studied cell configurations. Along the flow (A), facing the flow (F), the three cells clump (T) and the corner bridging cell (B). **Bottom right:** DAPI/Phalloidin staining of cells attached on scaffold struts.

At each of these locations, a spread out cell was positioned that conforms to the local geometry of the scaffold strut(s)–see Fig 5. The procedure that was used to obtain the detailed cell shapes is explained in the supplementary information. All cells were attached with discrete FAs which are located on the surface of the struts and of which the position did not change in time.

### Implementation

The Immersed Boundary implementation was realized using the Finite Element software FreeFEM++ [70], which solves the Stokes flow problem, with the Lagrangian forces computed in a coupled module implemented in the particle-based simulation platform Mpacts [71].

### Ethics statement

This procedure was approved by the ethics committee for Human Medical Research KU Leuven (ML7861). Patient informed written consent was provided by the legal guardian.

## Results and Discussion

Four potentially relevant mechanical measures were computed for cells experiencing flow conditions inside a scaffold pore: nodal displacement, cortical tension, normal pressure and local shear stress. Moreover, we compared between four different geometries as illustrated in Fig 5. All these geometries are regularly encountered in experimental set-ups where cells are attached to titanium scaffold struts—see Fig 5. A parameter study was performed to investigate the effect of changes in volumetric flow rate in the bioreactor on the specified mechanical measures and shows a linear relationship for all four quantities (see supplementary information). In the following section, we will discuss for each of these geometries the effect of flow on the four mechanical measures. In order to permit a direct comparison, the spatial distribution of every quantity will be shown for each geometry using the same scaling.

**Fig 6:**
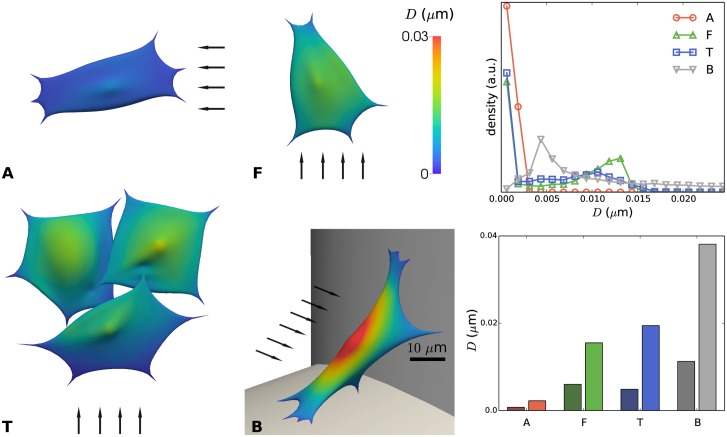
Displacement *D* of the immersed boundary due to flow, relative to the no-flow condition, for distinct configurations (A, F, T and B) of cells on scaffold struts. **Left**: color map showing local displacements. The arrows indicate the direction of the flow; scale bar 10 μm. **Top right**: distribution of the displacements for A, F, T, B. a.u.: arbitrary units. **Bottom right**: mean nodal displacement (dark color) and maximal nodal displacement for cases A, F, T and B.

Fig 6 summarizes the effect of flow on the nodal displacements. Very low displacements were obtained for flow parallel to the cylindrical strut (A), while intermediate displacements were found for flow perpendicular to the cylindrical strut (F) and relatively high displacements were observed for the cell forming a bridge at a scaffold corner (B).

For inlet bioreactor flow rates in the order of 1 ml/min, maximal displacements are in the range of 30 nm which has been reported as a critical displacement for the detachment of mesenchymal stem cells of bridged morphology from irregular scaffolds [72,73]. However, these studies were carried out using a one-way fluid–structure interaction (FSI) approach and did not consider the influence of cellular deformation on the surrounding fluid flow, something that in this study was included (two way interaction between cell and fluid). In another study [74], ‘microdisplacements by machine vision photogrammetry’ (DISMAP) was used to measure flow-induced strain of osteocytes on a flat substrate. Here, a linear strain-shear stress relationship was found. In our simulations, configurations (F) and (A) produce a maximal cell strain of resp. 0.412% and 0.062%, for empty scaffold shear stress magnitudes of resp. 0.0723 Pa and 0.0143 Pa. Using the linear relationship found in [74], strains of resp. 0.307% and 0.0608% would be expected. Even though the cell types are not identical, very similar values would be obtained by our computational model as found by these experiments. Moreover, the higher strain found in the (F) configuration is an expected result of the less shielded cell location.

The observed ranges of cell deformation are much smaller than typical deformations of cells in tissue: e.g. for chondrocytes in mature cartilage, strains higher than 20% (i.e. more than 1 μm) have been measured upon tissue compression [75]. However, it should be stressed that our simulations report the instantaneous elastic response in deformation to a step increase in flow velocity, and neglect the viscous deformations that might occur when cells are exposed to constant flow conditions for a long time (i.e. days).

**Fig 7:**
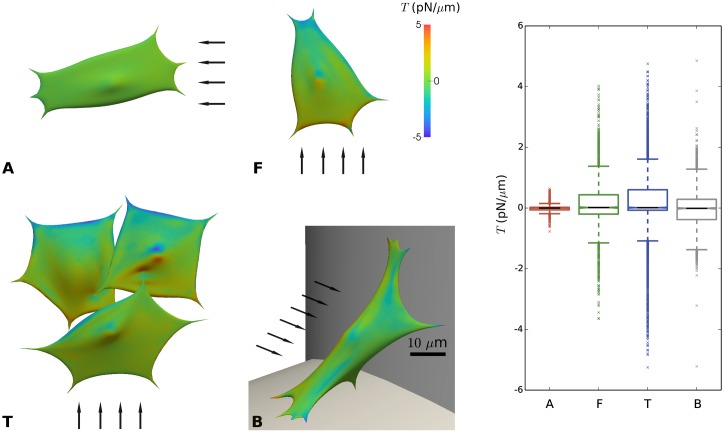
Cortical tension (T) due to imposed flow for distinct configurations (A, F, T and B) of cells on scaffold struts. Positive values indicate tensile conditions, while negative values indicate compressed conditions. **Left:** color map showing local tension. The arrows indicate the direction of the flow; scale bar 10 μm. **Right:** Boxplot showing distribution of local tension for cases A, F, T and B.

In Fig 7, the distribution of cortical tension *T* is shown for the four different geometrical configurations. Positive values of *T* indicate tensile conditions, whereas negative values of *T* indicate compressive stresses in the cortical shell. Unlike the cell deformations, which were maximal for the cell bridging between two struts (B), the maximal tension *T* occurs for cells on cylindrical struts with flow perpendicular to the strut (F) and (T). Moreover, maximal cortical tensions are observed close to the nucleus and close to FAs, with tensile stresses occurring at the side of incoming flow and compressive stresses at the side of out-going flow. Maximal tensions, which are highly localized, are around 5 pN/μm. For comparison, these values are several orders of magnitude below the values for membrane rupture [76]. Other experimental studies have looked at the induction of blebbing, for which it was reported that cortical tensions of at least 200 pN/μm were required [77], while inside blebs, cortical tensions between 10 and 100 pN/μm were measured [78]. The cell’s acto-myosin contractility alone creates an average resting cortical tension in the order of 0.5pN/μm [79]. In other words, the predicted additional cortical tension due to shear flow is relatively low, but it cannot be excluded that these tensions could nonetheless result in some conformational changes in the cytoskeleton.

**Fig 8:**
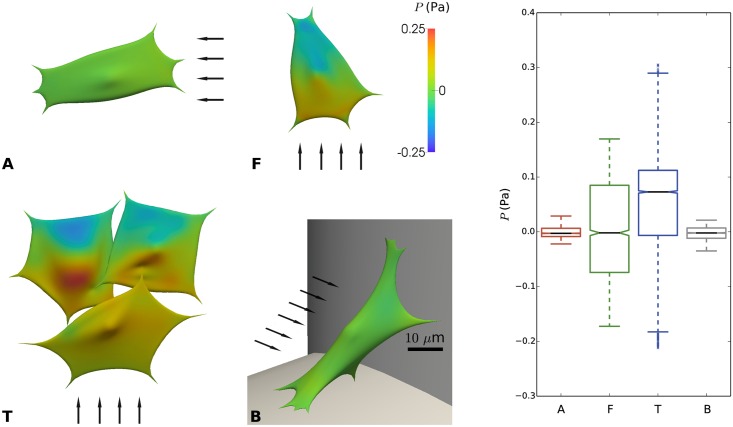
Pressure (P) on the cell surface due to imposed flow for distinct configurations (A, F, T and B) of cells on scaffold struts. **Left:** color maps showing local pressure. The arrows indicate the direction of the flow; scale bar 10 μm. **Right:** boxplot showing distribution of pressure for cases A, F, T and B.

Fig 8 shows the distribution of the fluid pressure (*P*) on each cell’s surface. Cells located in the configuration where the flow is facing the strut, i.e. where the flow velocity is the highest (F and T), show the largest variation in the pressure distribution, reaching a maximal amplitude of ± 0.5 Pa. Contrarily, cells located in (A) and (B) display few pressure differences due to the low flow speed and the lack of large changes in the flow streamlines.

**Fig 9:**
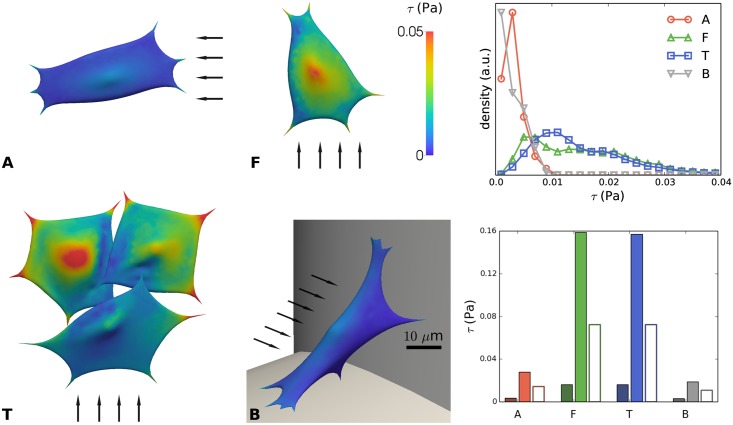
Shear stress (τ) on the cell surface due to imposed flow for distinct configurations (A, F, T and B) of cells on scaffold struts. **Left:** color maps showing local shear stress. The arrows indicate the direction of the flow; scale bar 10 μm. **Top right**: distribution of shear stress for A, F, T, B. **Bottom right**: mean shear stress (dark color) and maximal shear stress (light color) for cases A, F, T and B. The empty bars indicate the shear stress on the empty scaffold strut at the cell’s location (computed at the macro-scale).

Fig 9 shows the flow induced shear stress (*τ*) across the examined cells. While the average value of *τ* taken over the entire cell-surface (including the ‘bottom’ of the cells, facing the strut) is generally low due to the reduced flow speed and related shear stress at the bottom of the cell, it is very interesting to compare the maximum wall shear stress values at the top of the cells. Due to their location, configuration (F) and (T) show the highest shear stress, reaching up to 0.16 Pa while cells located in (A) and (B) show a low value of shear stress, around 0.02–0.03 Pa. *In vivo*, cells in the bone tissue have been found to experience shear stresses of 0.8–3.0 Pa during routine physical activity [41,80]. A maximal shear stress value of 0.77 Pa was observed on the surface of osteocytes due to flow in the pericellular domain [44].

Compared to the expected wall shear stresses in an empty scaffold, we see that the maximum stress predicted by the IBM is generally about twice as high, while the average stress over the complete cell surface is significantly lower. This clearly indicates the importance of taking the shape and mechanical response of the cells into account for estimating their relevant wall shear stress. Regarding the (F) and (T) configurations, as expected, the distribution of shear stress is more homogenous with half of the cell surface exposed to a higher value than 0.015 Pa, while the two other configurations present most of their surface exposed to low shear stress value (below 0.005 Pa). For the flow rate level used in this study, we have previously reported the effect of increasing flow rates resulting in osteogenic priming of hPDCs in the absence of supplementary growth factors [8] with genes such as osterix and bone sialoprotein being slightly upregulated. Additionally, again for similar flow rates and in the presence of osteoinductive medium, hPDCs have been shown to secrete higher levels of ECM and to enhance mineralization for increasing flow rates [81]. It has been observed [82] that for a shear stress value exceeding a threshold value of 0.088 Pa, human MSCs can detach from the scaffold surface and by doing so negatively affect the final properties of tissue engineered constructs, resulting in an inhomogeneous distribution of neotissue across the scaffold [83]. This illustrates the importance of having a numerical model such as the one presented in this study, to quantify and characterize the mechanical environment that cells experience in novel scaffold designs in order to avoid the development of suboptimal or detrimental mechanical regimes.

### Conclusions and outlook

In the presented work, a novel application of the immersed boundary method was developed, representing a deformable cell exposed to microscopic flow and attached to a 3D scaffold inside a perfusion bioreactor. Cells were represented by a deformable Lagrangian surface mesh, which was immersed in an Eulerian fluid domain, with flow in the Stokes regime. We demonstrated the effect of shear flow for multiple realistic geometrical cell configurations and strut locations inside a regular pore scaffold. This tool can be used to estimate shear flow conditions directly on the surface of individual cells, and assess the micro-scale variability of mechanical conditions inside single scaffold pores. The instantaneous cell stiffness was measured using AFM experiments on hPDCs, and the mechanical model was calibrated using micro-pipette aspiration simulations in the range of short term deformations. Simulations confirmed that mechanical cues originating from the flow are highly dependent on the exact geometry of the cell and its environment. For example, a cell on a cylindrical strut with flow perpendicular to the strut will experience a much larger shear stress than a cell on a similar strut with parallel flow. This should be a major consideration when designing novel scaffold designs. Moreover, it was found that wall shear stress calculated in the empty scaffold would underestimate the actual maximal wall shear stress experienced by the cells by a factor of two in the investigated cases.

Next, the model was used to estimate the additional instantaneous flow-induced cell deformation, tension and pressure. Compared to the cell-generated deformation and tension due to acto-myosin activity these values are very small for the applied realistic flow conditions. For example, the predicted magnitude of additional cortical tension due to flow is much lower than the pre-stress of a spread out cell. Hence, it is not expected to affect the tensional homeostasis of a cell in steady-state conditions. Nonetheless, in cyclic conditions, small deviations from the resting state can still have pronounced biological effects, as is explained by the tensegrity model [32,33]. In the same vein, small cytoskeletal deformations away from the cell's resting configuration might alter the configuration of several intracellular mechanosensing molecules, which are linked to downstream targets in pathways such as the mitogen-activated protein kinase (MAPK) or phosphoinositide-3-kinase (PI3K) pathways [84]. Since the predicted strain of the cell is highest near its attachment points on the substrate, which also constitute focal sites of mechanotransduction machinery, a discernible biological response could result even from small deformations, as long as the dynamics of the perturbations in flow conditions are faster than the relaxation time of tensional homeostasis.

In the current work, a strongly simplified model for the mechanical behavior of single cells was used, which limits its predictive value to small deviations from the resting state. A more in-depth computational analysis of the mechanisms at play involved in large cell deformations would require a more detailed model of the mechanoadaptive behavior of the cytoskeleton and will be reserved for future research. Similarly, the effect of shear flow on the long timescale viscous-like deformation of living cells remains to be investigated. This would also require a more elaborate description of the cell’s mechanical behavior, which for this study was greatly simplified and limited to linearly elastic deformation. The model’s limitation of small deformations and short timescales will mainly restrict in scope the predictions of cell deformation and cortical tension, whereas predictions of shear stress and local pressure, being surface properties, are expected to remain largely unaffected. Finally, to simulate adhesion and detachment behavior (e.g. in very high shear flows), the presented methodology has to be extended since adhesion is only implicitly captured by placing FAs out of the fluid domain thereby fixing them in space independently of applied forces. For this, an adhesion force formulation as proposed in [62] could be included.

A parameter study (see supplementary information) showed linear behavior in the relevant cell-mechanical and flow parameters, showing that the model can be used to inter-/extrapolate to different cell types and flow conditions. For a cell of thickness 5 μm facing flow on a strut of diameter 200 μm, the wall shear stress can be estimated as: τ ≈ 0.08·*Q*_*in*_. Evidently, the maximal wall shear stress experienced by a cell does not depend strongly on the cell’s mechanics. This implies that even though a cell may undergo structural changes (e.g. migration, re-alignment), it can still reliably ‘sense’ the shear flow (e.g. with its primary cilium). This study constitutes an important step towards model-based control of a cell’s biophysical micro-environment (stem cell niche engineering) in a perfusion bioreactor.

## Supporting Information

S1 Text Computational and experimental implementation details. Description of the methodology to obtain computational meshes for realistic spread out cells, the technical procedure of the AFM experiments, and numerical details of the CFD simulations.
(DOCX)

S1 Fig Geometrical model of the cell. **From left to right:** An example of the procedure for obtaining geometries of cells attached in flow, starting from a perfect sphere. The blue sphere inside the cell represents the nucleus.
(TIFF)

S2 Fig Additional data on AFM experiments. a) Selection of regions on the cellular extension used for the cortex-stiffness analysis, b) Average thickness h of all thus selected regions, c) Young's modulus E_c_ vs. thickness h for all regions, no correlations are apparent.
(TIFF)

S3 Fig Results of a parameter study varying inlet volumetric flow rate Q_in_ and cortex stiffness k_s_ for configuration (F). **Top left:** maximal local displacement; **Top right:** maximal normal pressure; **Bottom left**: maximal local shear stress and **Bottom right**: maximal local tension. From *Q*_*in*_ the Dirichlet boundary conditions in the micro-scale model were determined using a CFD simulation of the complete scaffold pore—Fig 4A. The resulting maximal deformation, pressure, shear stress and cortical tension were quantified. One might notice that the dependence on *Q*_*in*_ is linear, which is due to the Stokes’ flow regime, which is valid for the investigated range of flow rates. Except for the maximal deformations, the effect of the cells’ stiffness is very small.
(TIFF)

S4 Fig Slice at *x* = 0 through the flow domain of configuration ‘F’—see Fig 5—with the color scale indicating the magnitude of the flow velocity, for varying levels of Eulerian mesh refinement. The Eulerian mesh is characterized by the average strut size, *L*_*e*_ which is varied between 500 nm and 2000 nm.
(TIFF)

S5 Fig Fluid velocity profile in the y-direction obtained in a central region in the *y* and *z* dimension (see S4 Fig), at the location of a spread-out cell in configuration “F”, for varying levels of Eulerian mesh refinement. At each height, an average was taken over a narrow region of *x* ∈ [-5 μm, 5 μm] and *z* ∈ [-5 μm, 5 μm].
(TIFF)

S6 Fig Node displacement of the Lagrangian mesh (representing the cell) in the ‘F’ configuration for varying levels of Eulerian mesh refinement. If the Lagrangian mesh is much finer than the Eulerian grid, the Immersed Boundary Method will fail to properly resolve internal tensions, and an incorrect result for the cell displacement will be obtained.
(TIFF)

S7 Fig Standard deviation of the nodal displacement (see S6 Fig) as a function of the mean edge length *L*_*e*_ of the Eulerian grid (representing refinement level), for a Lagrangian mesh size with an average resting length of *L*_*l*_ = 679nm. When *L*_*e*_ is much larger than *L*_*l*_, an incorrect solution for the mechanical response of a cell can be expected.
(TIFF)

S1 Table Comparison of boundary condition velocities on the micro-scale systems obtained from a CFD simulation of a complete scaffold pore, both using Navier-Stokes and Stokes Equations. (XLS)

S1 Data AFM measurement data, provided as Paraview readable files containing height information of the measured cells, as well as cantilever deflection and statistics used for estimating the local Young’s modulus. (ZIP)

S2 Data Simulation scripts and computational meshes. Python scripts used for simulating the Immersed Boundary interactions using Mpacts, and FreeFEM++ scripts for computing the solution for Stokes’ flow. The geometries of the cells in the various configurations (A, F, T, B) are provided as.obj files.
(ZIP)

S3 Data Results for micropipette aspiration simulations, provided as a text file (‘results.csv’) with comma separated values, with columns containing resp. *k*_*c*_, *t*_*c*_, *L*_*p*_/*R*, *k*_*b*_, *E*_*∞*_, *L*_*p*_, *E*_*c*_, with symbols as used above.
(ZIP)

S4 Data Results for IBM simulations, containing Paraview readable output files reproduced in Figs 6–9, and Python scripts which were used to read and plot these data. (ZIP)

S5 Data Results for SI parameter study (S3 Fig), provided as a text file (‘results.csv’) with comma separated values, in which each row represents the results from a single simulation with parameters and output measures as explained in the file header. (ZIP)
