# Supplementary material for: Immersed Boundary Models for Quantifying Flow-Induced Mechanical Stimuli on Stem Cells Seeded on 3D Scaffolds in Perfusion Bioreactors
Source: PLoS Comput Biol. 2016 Sep 22;12(9):e1005108. doi: 10.1371/journal.pcbi.1005108 (PMC5033382; doi:10.1371/journal.pcbi.1005108)
Supplement: S1 Text — Description of the methodology to obtain computational meshes for realistic spread out cells, the technical procedure of the AFM experiments, and numerical details of the CFD simulations. (DOCX) [file pcbi.1005108.s001.docx]

**S1 Text. Immersed Boundary Models for quantifying flow-induced mechanical stimuli on stem cells seeded on 3D scaffolds in perfusion bioreactors**

Y. Guyot^1,2*^, B. Smeets^1,3,5*^, T. Odenthal^3^, R. Subramani^3^, F. P. Luyten^1,4^, H. Ramon^3#^, I. Papantoniou^1,4#^, L. Geris^1,2,5#^

^1^ Prometheus, Division of Skeletal Tissue Engineering, KU Leuven, Onderwijs en Navorsing 1(+8), Herestraat 49 - PB813, B-3000, Leuven, Belgium.

^2^ Biomechanics Research Unit, Université de Liège, Chemin des Chevreuils 1 - BAT 52/3, B-4000 Liège, Belgium

^3^ Division of Mechatronics, Biostatistics and Sensors (MeBioS) Kasteelpark Arenberg 30 - box 2456 3001 Leuven, Belgium.

^4^ Skeletal Biology and Engineering Research Center, KU Leuven, Onderwijs en Navorsing 1(+8), Herestraat 49 - PB813, B-3000 Leuven, Belgium.

^5^ Biomechanics Section, KU Leuven, Celestijnenlaan 300C – PB 2419, B-3001 Leuven, Belgium

**Initialization of the cell geometry**

In order to obtain realistic shapes of spread-out and stretched-out cells on cylindrical scaffold struts, we perform a separate relaxation simulation. Hereto we start with a spherical geometry, generated from a subdivided icosahedron, and slowly translate pre-selected adhesion points towards specific attachment points on the scaffold, which are obtained by displacing the nodes normal to the sphere’s surface and subsequently projecting them on the scaffold surface – S1 Fig. During this process, the ‘free’ nodes of the cell relax towards an energy-minimizing equilibrium. The node displacements are calculated in the absence of flow, by solving for the velocity $\boldsymbol{v}\left( s,t \right)$:

|  | $\boldsymbol{\Gamma}\cdot\boldsymbol{v}\left( s,t \right)=\boldsymbol{f}\left( s,t \right)$, |  |
| --- | --- | --- |

in which $\boldsymbol{f}\left( s,t \right)$are the internal mechanical forces and $\boldsymbol{\Gamma}$ is an arbitrary friction matrix. It is clear that this does not simulate the way that real cells obtain their spread out configuration, but it should be pointed out that the fundamental mechanisms of protrusions, contractility and maturing focal adhesions will produce very similar shapes with elongated, curvature minimizing surfaces.

**Atomic Force Microscopy procedure**

The complete selection procedure consists of the following steps [1]:

1. Fit Eq. (14) to all the force-distance curves obtained on the cell (typically 16×16 or 32×32). To obtain a robust fit, it has been proven beneficial to estimate the contact point ($\delta=0$) at the same time as the apparent cortical Young’s modulus.
2. Select thin extensions of the cell.
3. Calculate local slopes of the smoothed surface and reject angles with respect to the vertical > 20 degrees to minimize artifacts due to tip-sliding or lateral cell movement.
4. Reject bad fits based on a $\chi^{2}$-statistic (rare).

The remaining apparent cortical Young’s moduli are averaged per patch on the cell and the distribution of these is shown in Fig. 2d, the global average over all measured cells and all patches is 3.5 ± 2 kPa.

**Mesh independence and numerical details.**

CFD simulations were performed on an Eulerian mesh with a mesh size of 1μm and a total of 250k-350k tetrahedrons. In order to solve the constitutive equations, a finite element scheme was used with P2 type elements for the velocity – required for a stable evaluation of the shear stress from the gradient of the velocity - and P1 elements for the pressure. In order to compute the Reynolds number we can use:

$$\mathrm{Re}= \frac{\rho vD_{H}}{\mu},$$

with$D_{H}$ the hydraulic diameter:

$$D_{H}= \frac{4A}{P},$$

with cross-sectional area *A* and cross-sectional wet circumference *P.* In the ‘worst-case’ configuration ‘F’, we arrive at $D_{H}$ = 27 μm. For a fluid density of 1000 kg/m^3^, a maximal velocity (boundary condition) of 1.2 mm/s and the viscosity of water as 85 mPa∙s, we obtain a Reynolds number of Re = 0.039 $\ll$ 1. In other words, the Stokes Regime is applicable for solving the flow conditions in the micro-scale problem.

For the simulations on a whole scaffold pore, which were performed to obtain the boundary conditions for the microscopic systems, the hydraulic diameter *D_H_ =* 400μm, and a flow rate was set of 1ml/min. Hence, Re = 0.56. Although this value is still smaller than one, it makes sense to compare it to a full Navier-Stokes simulation, to ensure that the approximation of the Stokes Regime is appropriate. For comparison, the boundary conditions of our microscopic systems, obtained from a simulation on a complete scaffold pore, are listed in S1 Table, computed with both Stokes and Navier-Stokes equations. As can be seen, the maximal difference in the obtained values is 0.3%, which is still very small, and is not expected to influence the results of the predicted quantities.

In order to examine the dependence of the calculations on the computational grid, we examined the fluid flow in cell configuration ‘**F**’ – see Fig. 5 - for four different levels of mesh refinement, varying the average edge length of the Eulerian (*L_e_*) mesh between 500 and 2000 nm, see S4 Fig.

S5 Fig. shows the magnitude of the fluid flow velocity as a function of the height (y-axis) in the center of the computational domain, i.e. at the location of the submerged cell. When refining the Eulerian mesh, the velocity magnitudes converge to a stable profile. From this study, it was concluded that an Eulerian mesh size of *L_e_*=1000 nm is sufficiently fine to obtain a grid-independent solution.

S6 Fig. shows the local displacements of the cell surface in configuration ‘**F**’ for different levels of refinement of the Eulerian mesh, again varying *L_e_* between 500 and 2000 nm. When the grid size is large compared to the size of the Lagrangian mesh (the mean edge length of the deformable cell *L_l_* =679 nm), the IBM coupling fails to provide a correct solution, since tensions existing between adjacent nodes in the Lagrangian mesh are not adequately relaxed. The same can be seen in S7 Fig., which shows the standard deviation of the nodal displacements for varying *L_e_*.

**References**

[1] Rico F, Roca-Cusachs P, Gavara N, Farre R, Rotger M, et al. (2005) Probing mechanical properties of living cells by atomic force microscopy with blunted pyramidal cantilever tips. Phys Rev E Stat Nonlin Soft Matter Phys 72: 021914.
