# Supplementary figures and images for: Immersed Boundary Models for Quantifying Flow-Induced Mechanical Stimuli on Stem Cells Seeded on 3D Scaffolds in Perfusion Bioreactors
Source: PLoS Comput Biol. 2016 Sep 22;12(9):e1005108. doi: 10.1371/journal.pcbi.1005108 (PMC5033382; doi:10.1371/journal.pcbi.1005108)

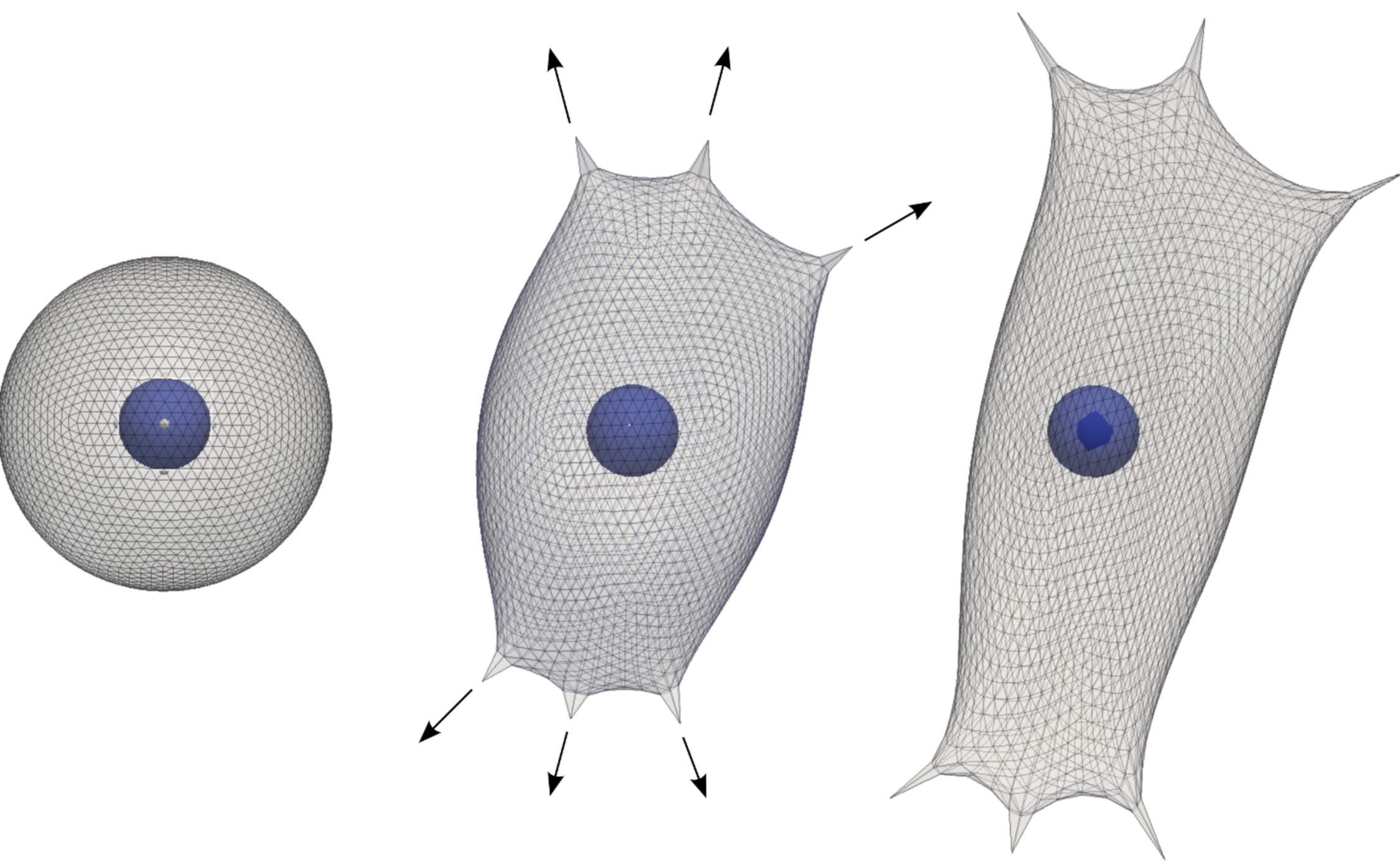

Supplement: S1 Fig — From left to right: An example of the procedure for obtaining geometries of cells attached in flow, starting from a perfect sphere. The blue sphere inside the cell represents the nucleus. (TIFF) [file pcbi.1005108.s002.tiff]

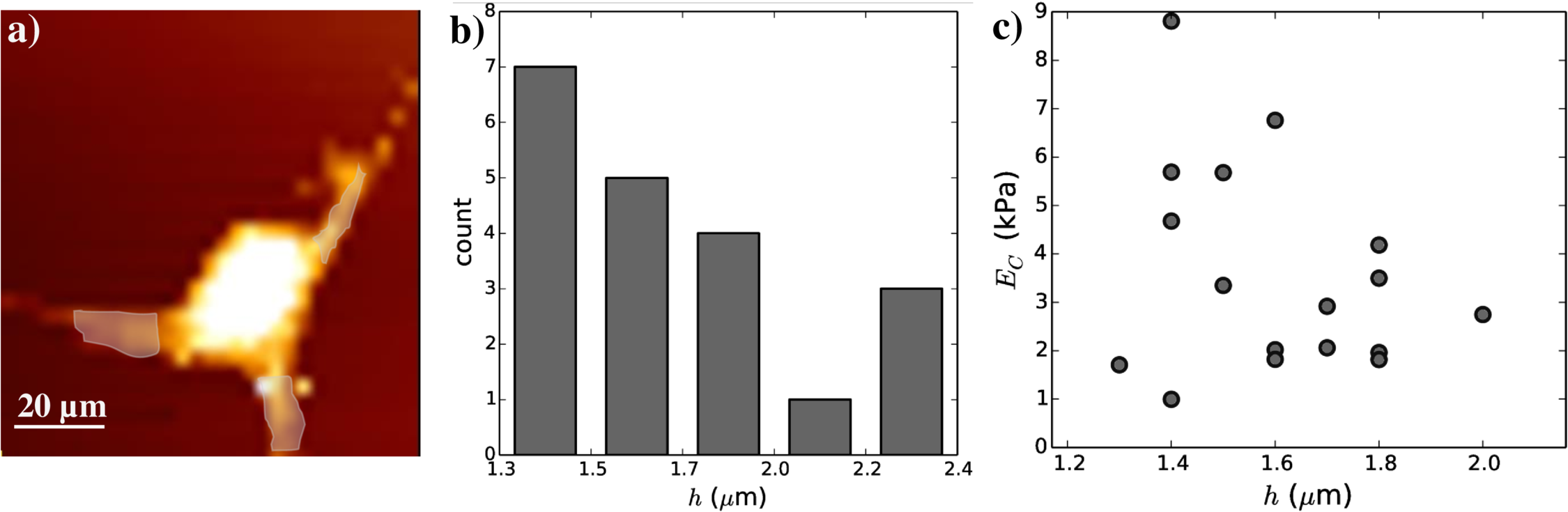

Supplement: S2 Fig — a) Selection of regions on the cellular extension used for the cortex-stiffness analysis, b) Average thickness h of all thus selected regions, c) Young's modulus Ec vs. thickness h for all regions, no correlations are apparent. (TIFF) [file pcbi.1005108.s003.tiff]

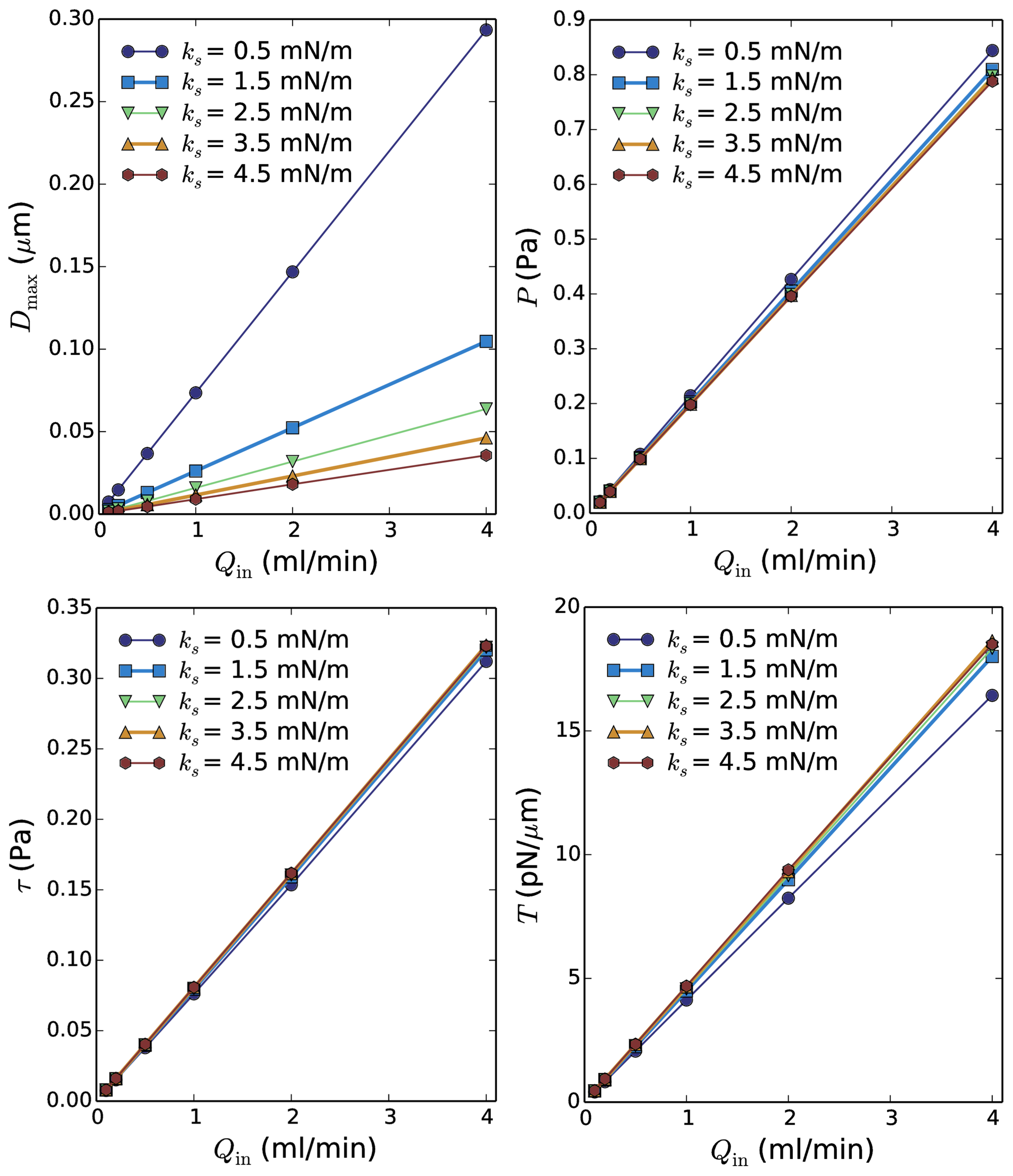

Supplement: S3 Fig — Top left: maximal local displacement; Top right: maximal normal pressure; Bottom left: maximal local shear stress and Bottom right: maximal local tension. From Qin the Dirichlet boundary conditions in the micro-scale model were determined using a CFD simulation of the complete scaffold pore—Fig 4A. The resulting maximal deformation, pressure, shear stress and cortical tension were quantified. One might notice that the dependence on Qin is linear, which is due to the Stokes’ flow regime, which is valid for the investigated range of flow rates. Except for the maximal deformations, the effect of the cells’ stiffness is very small. (TIFF) [file pcbi.1005108.s004.tiff]

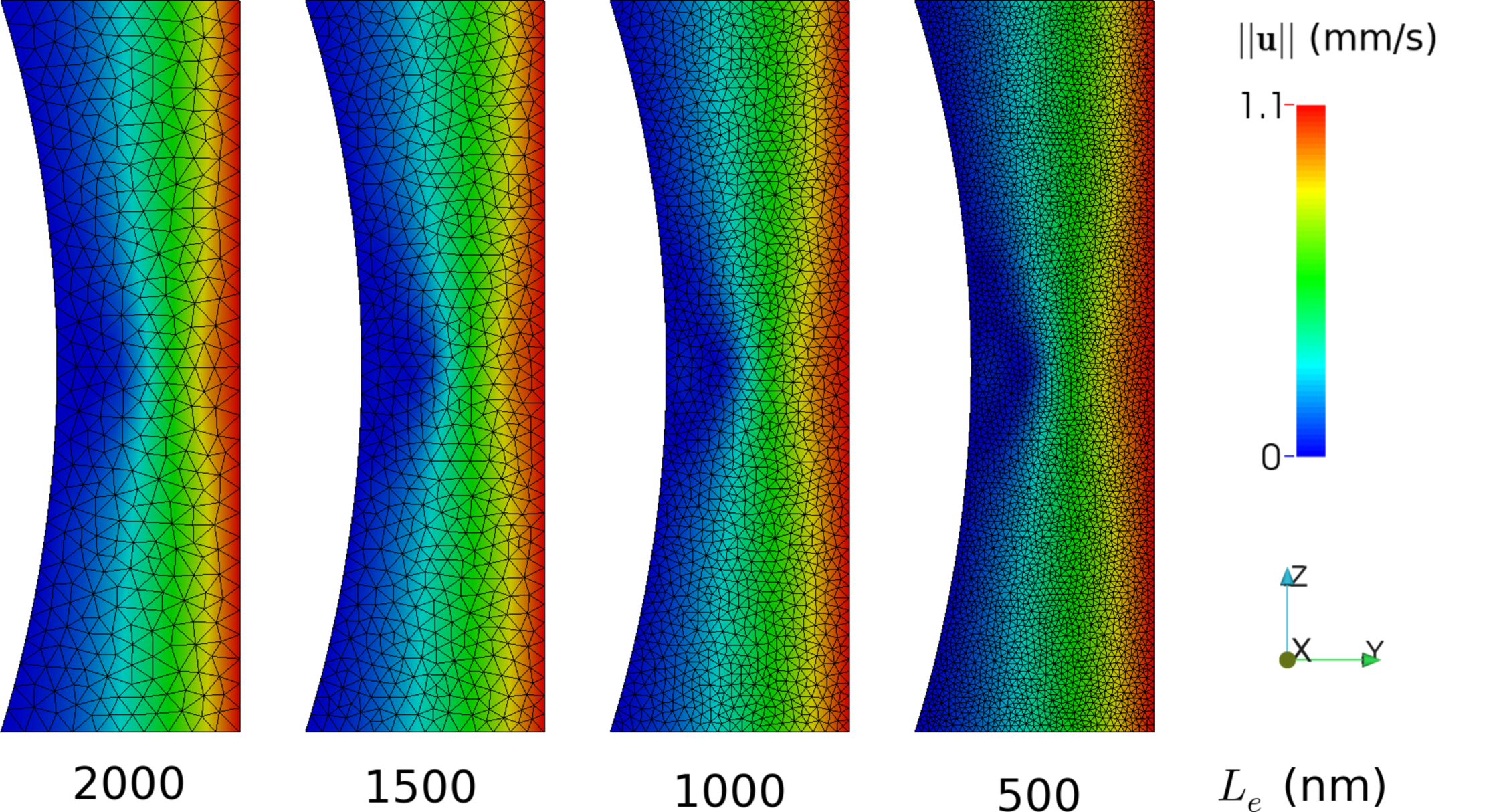

Supplement: S4 Fig — The Eulerian mesh is characterized by the average strut size, Le which is varied between 500 nm and 2000 nm. (TIFF) [file pcbi.1005108.s005.tiff]

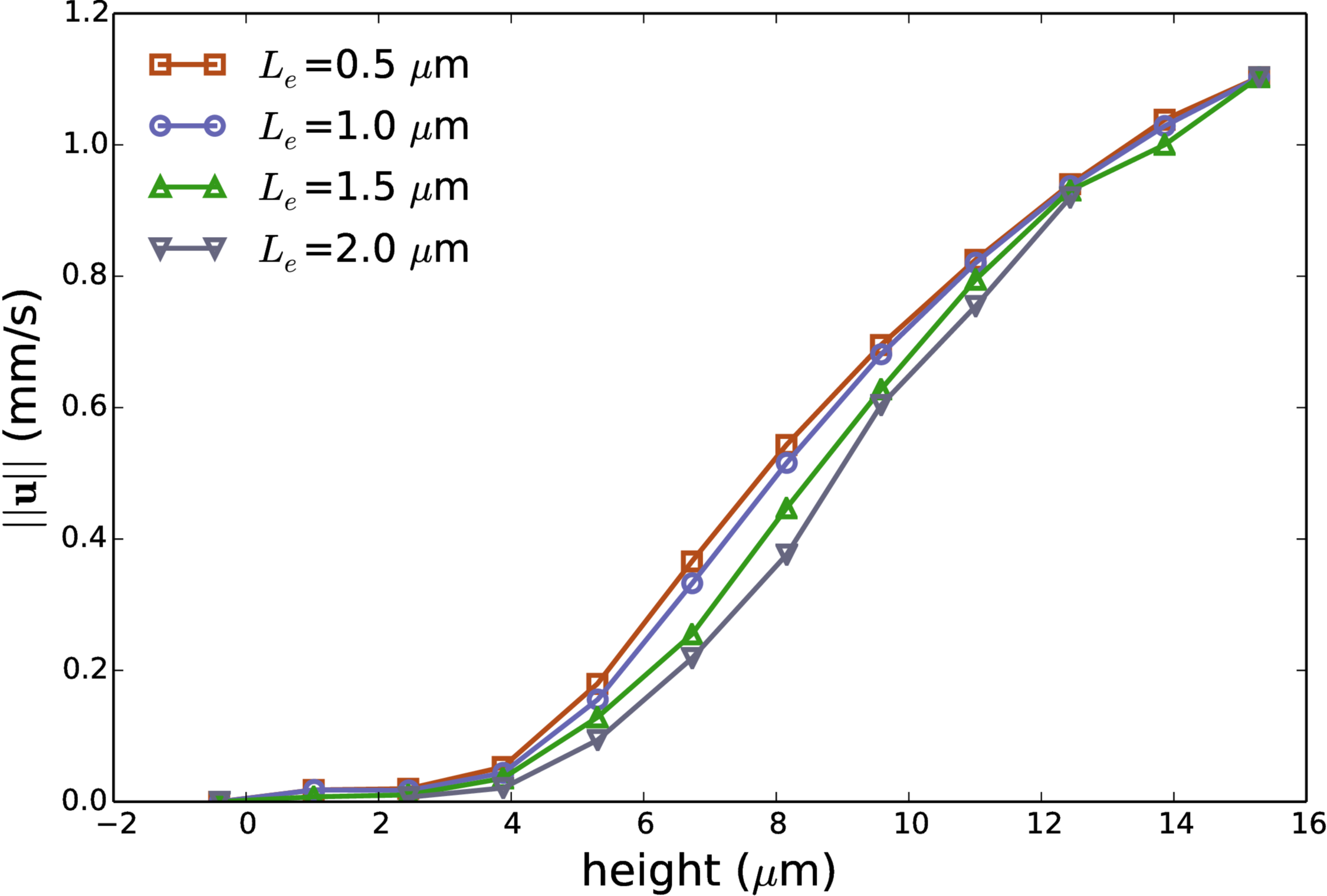

Supplement: S5 Fig — At each height, an average was taken over a narrow region of x ∈ [-5 μm, 5 μm] and z ∈ [-5 μm, 5 μm]. (TIFF) [file pcbi.1005108.s006.tiff]

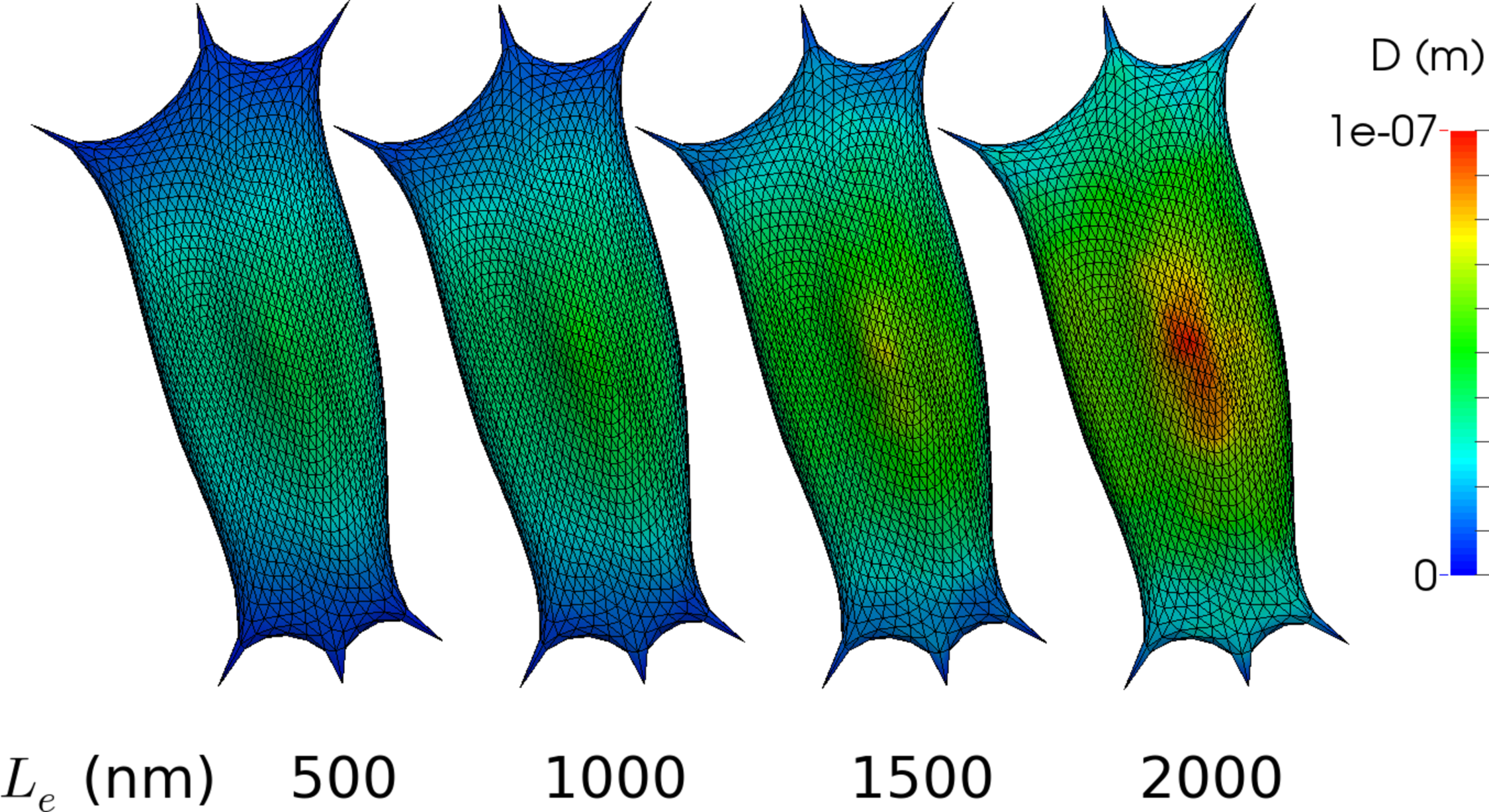

Supplement: S6 Fig — If the Lagrangian mesh is much finer than the Eulerian grid, the Immersed Boundary Method will fail to properly resolve internal tensions, and an incorrect result for the cell displacement will be obtained. (TIFF) [file pcbi.1005108.s007.tiff]

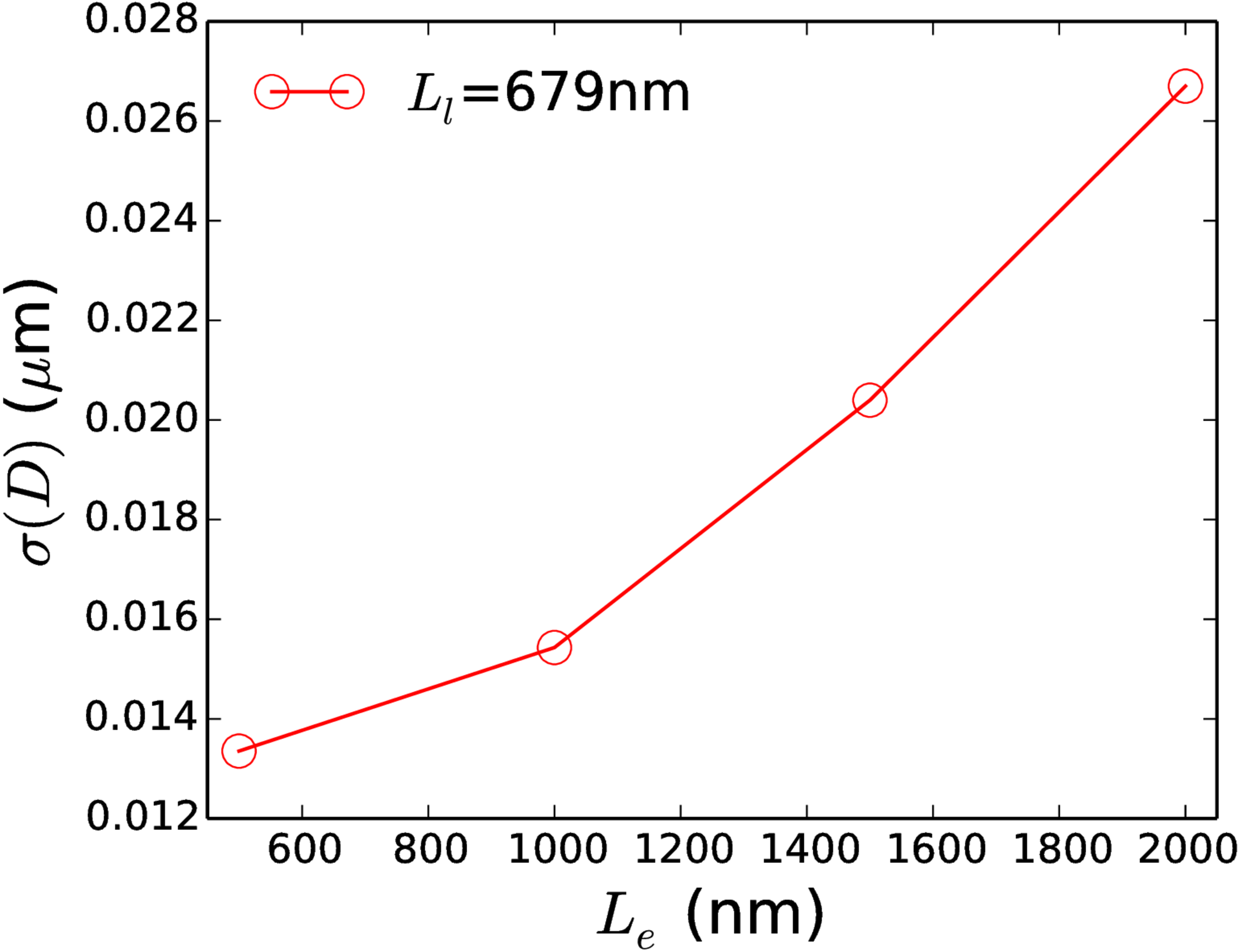

Supplement: S7 Fig — When Le is much larger than Ll, an incorrect solution for the mechanical response of a cell can be expected. (TIFF) [file pcbi.1005108.s008.tiff]

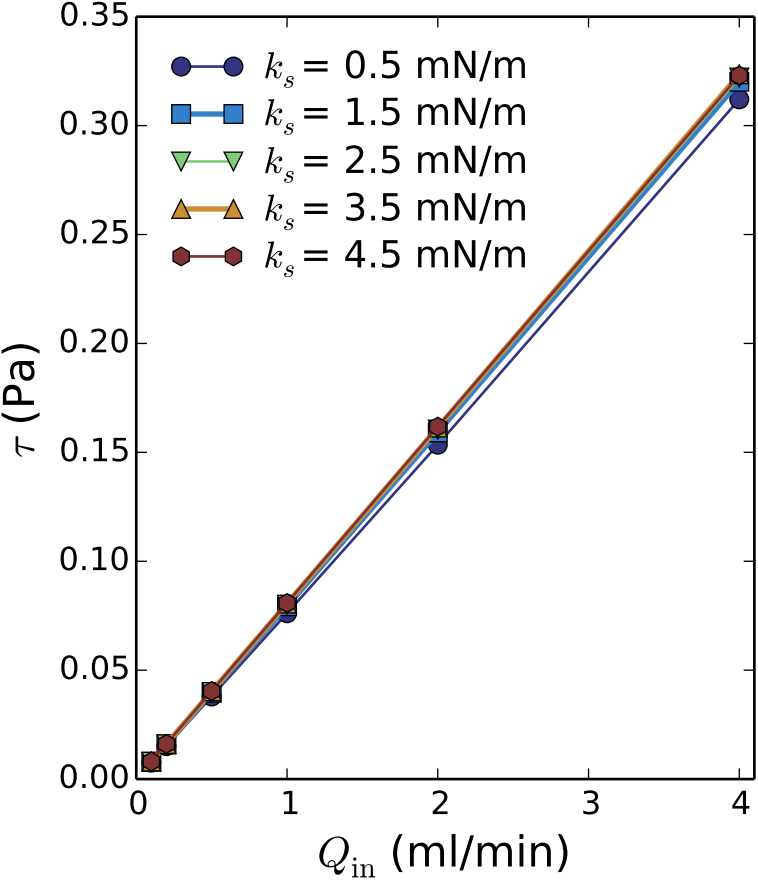

Supplement: S5 Data — (ZIP) [file pcbi.1005108.s014.zip › pstudy_flowrate_kcortex/lines_tau_qin_varks.pdf]

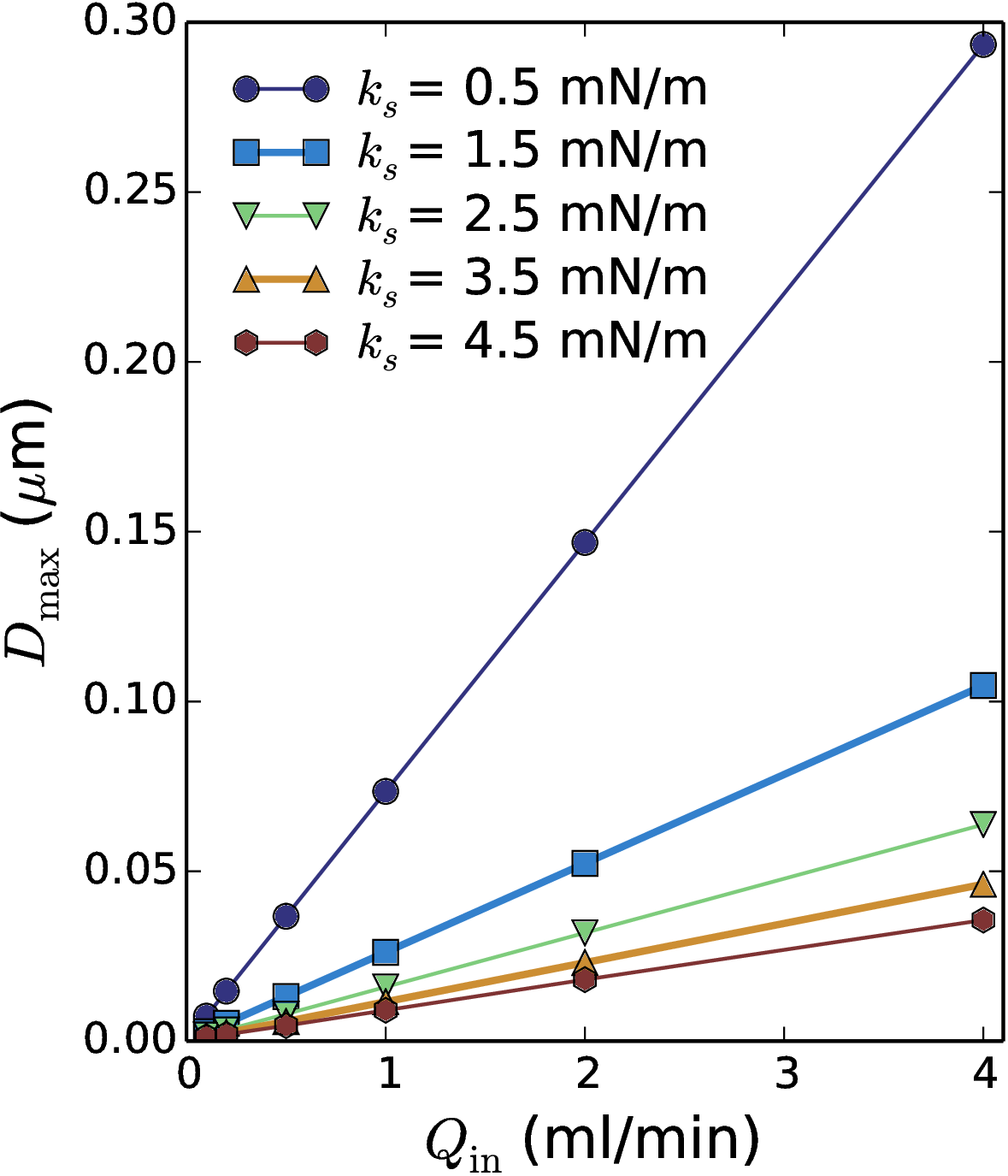

Supplement: S5 Data — (ZIP) [file pcbi.1005108.s014.zip › pstudy_flowrate_kcortex/lines_deltamax_qin_varks.png]

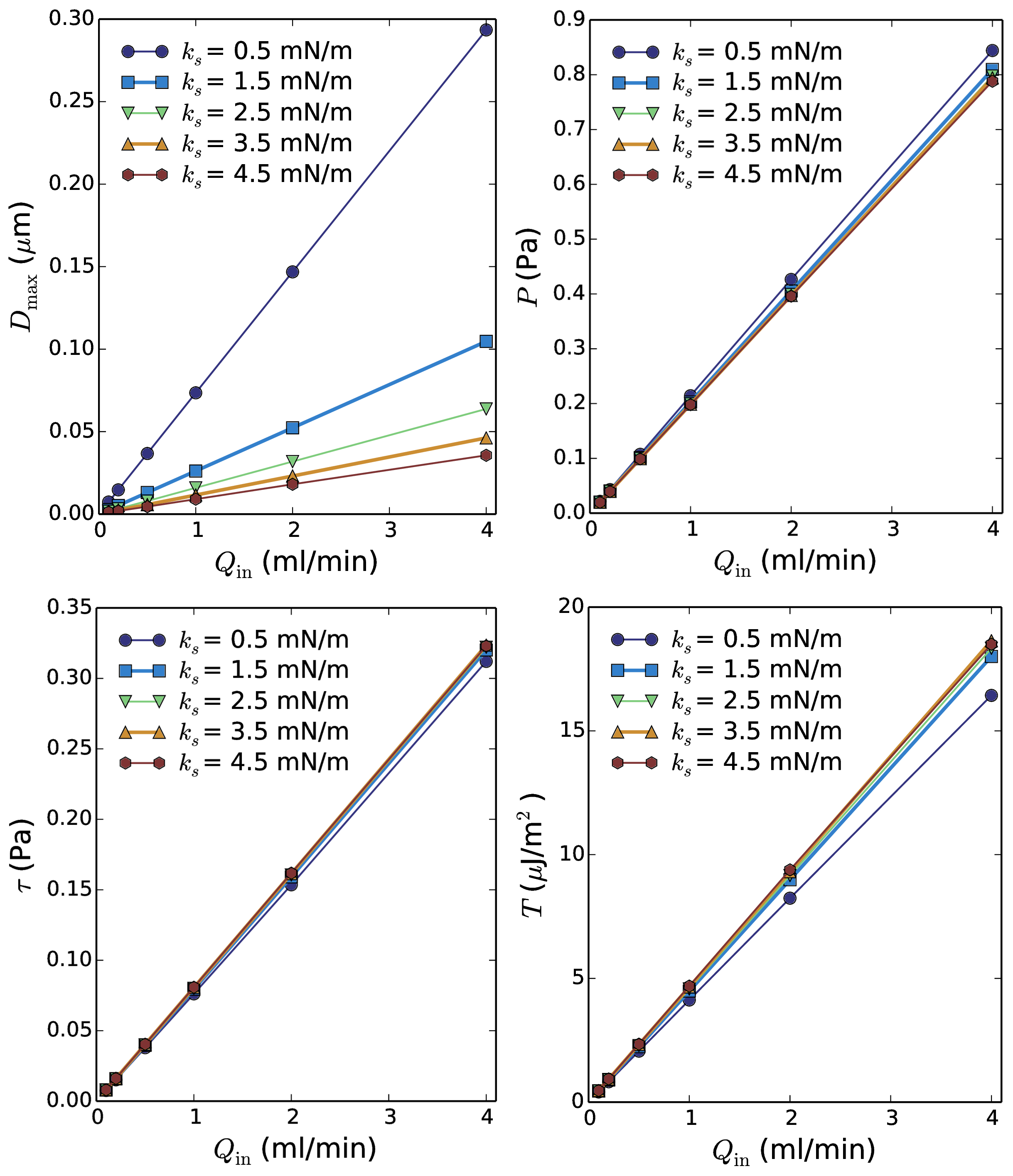

Supplement: S5 Data — (ZIP) [file pcbi.1005108.s014.zip › pstudy_flowrate_kcortex/montage_lines.png]

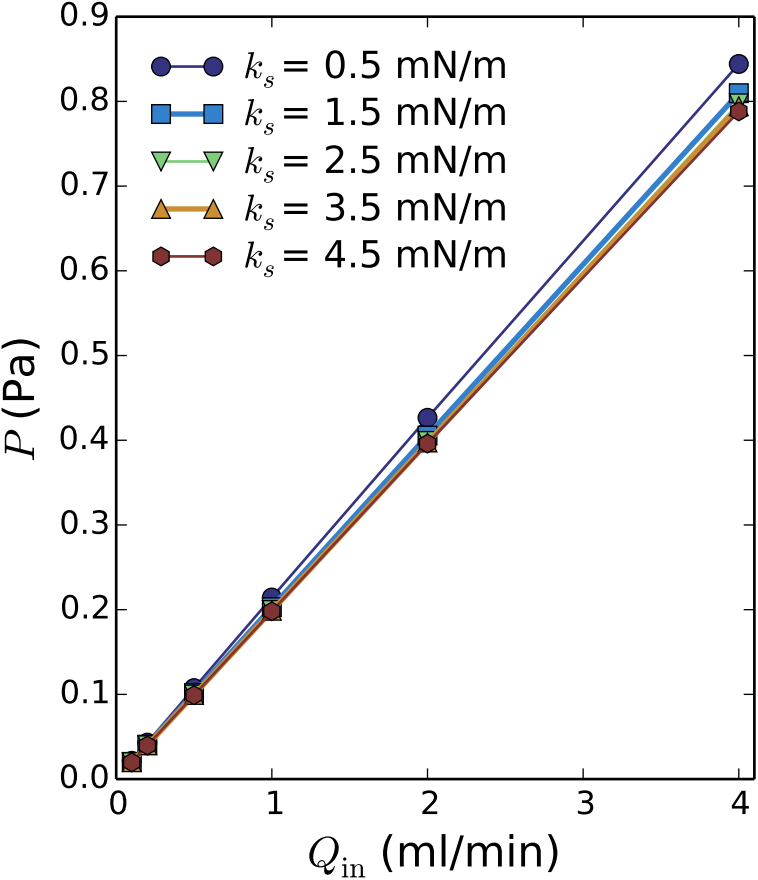

Supplement: S5 Data — (ZIP) [file pcbi.1005108.s014.zip › pstudy_flowrate_kcortex/lines_pressure_qin_varks.pdf]

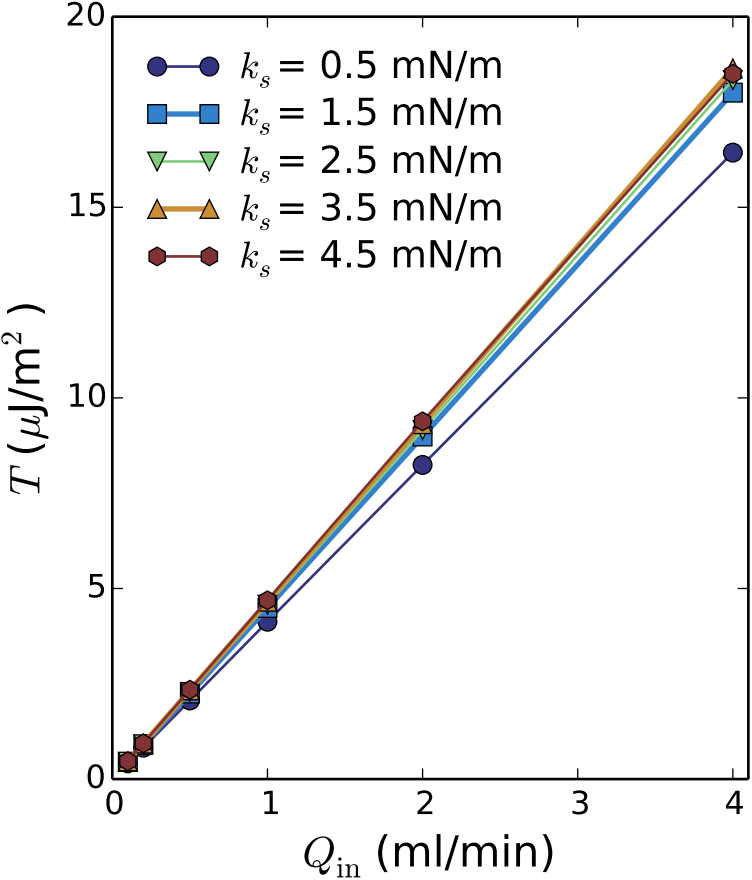

Supplement: S5 Data — (ZIP) [file pcbi.1005108.s014.zip › pstudy_flowrate_kcortex/lines_tension_qin_varks.pdf]

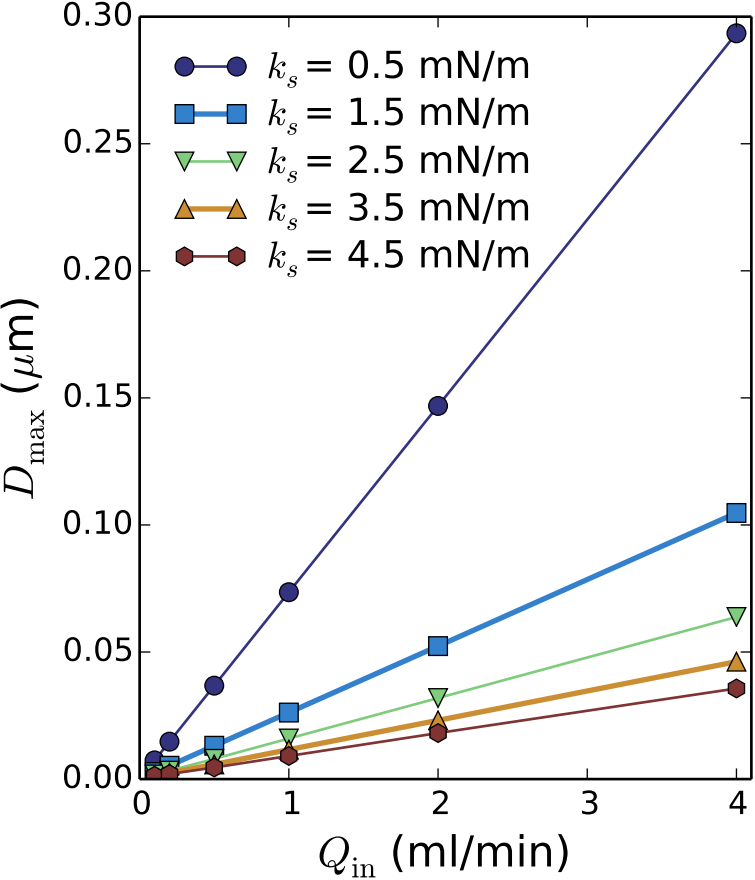

Supplement: S5 Data — (ZIP) [file pcbi.1005108.s014.zip › pstudy_flowrate_kcortex/lines_deltamax_qin_varks.pdf]

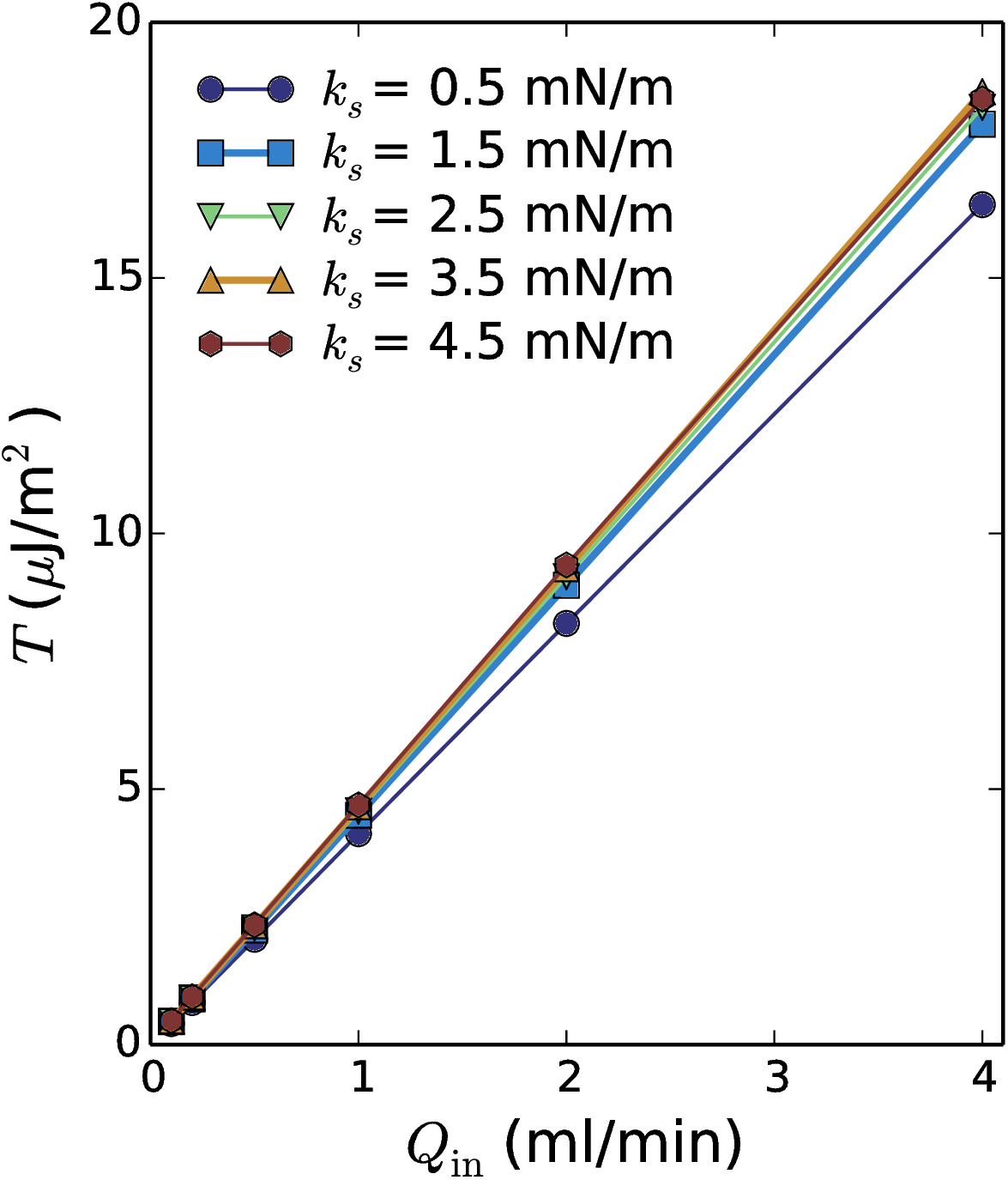

Supplement: S5 Data — (ZIP) [file pcbi.1005108.s014.zip › pstudy_flowrate_kcortex/lines_tension_qin_varks.png]

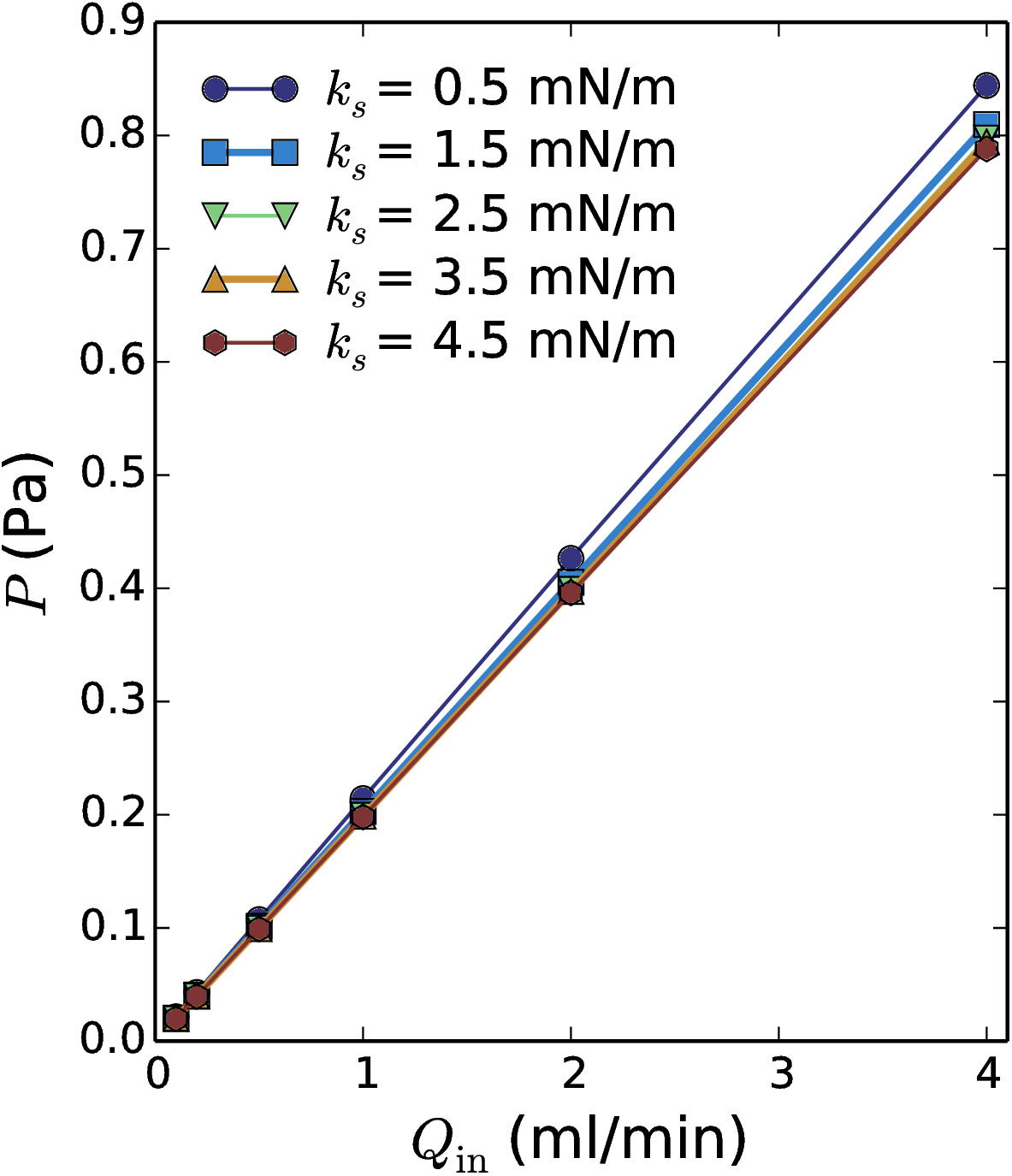

Supplement: S5 Data — (ZIP) [file pcbi.1005108.s014.zip › pstudy_flowrate_kcortex/lines_pressure_qin_varks.png]

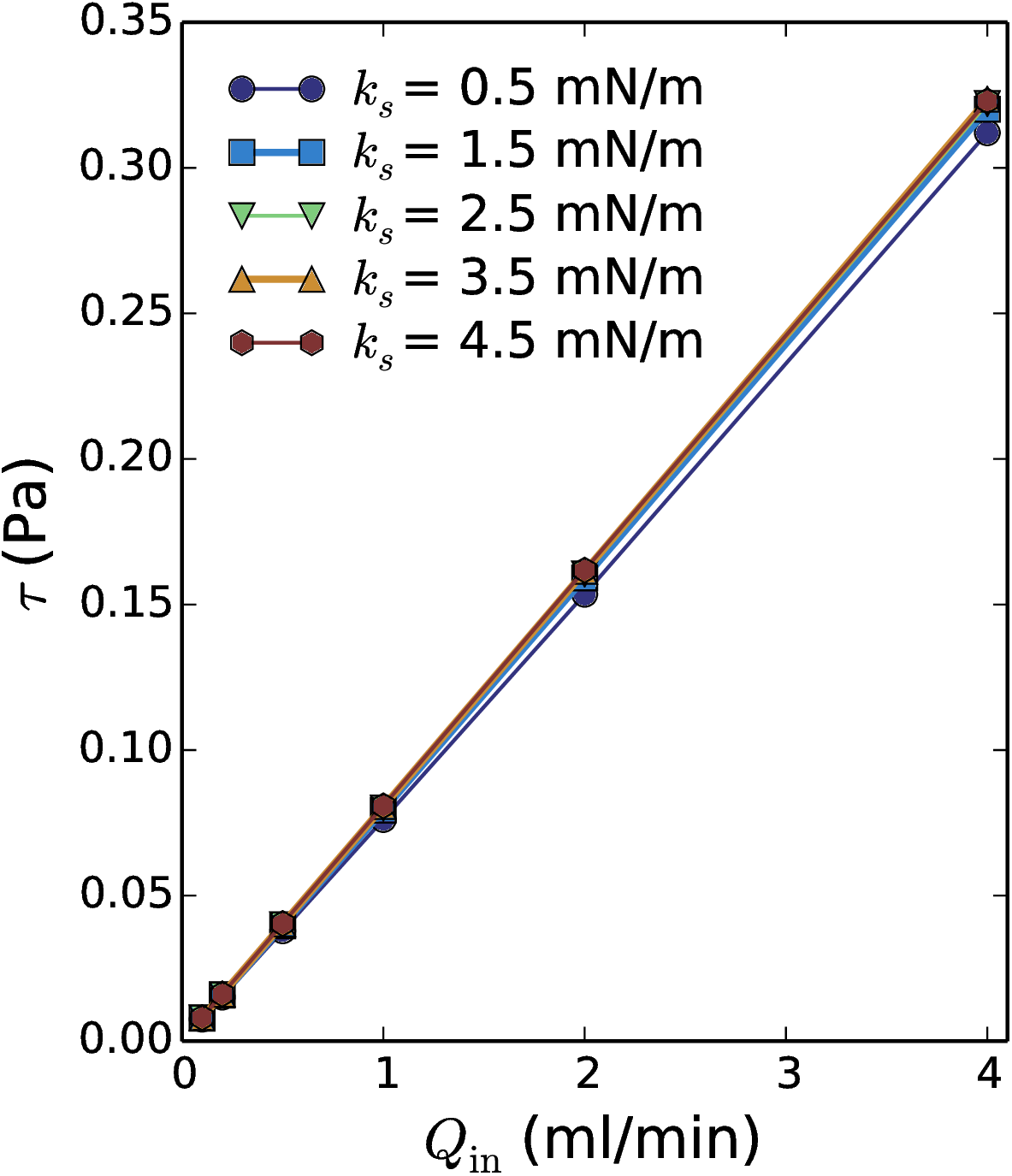

Supplement: S5 Data — (ZIP) [file pcbi.1005108.s014.zip › pstudy_flowrate_kcortex/lines_tau_qin_varks.png]
